# Examining Early Vocabulary Growth Trajectories in Late Talkers in a Low‐Income Longitudinal Sample

**DOI:** 10.1111/infa.70036

**Published:** 2025-08-07

**Authors:** Britt Singletary, Hui Jiang, Winifred Graham Wilberforce, Daniela Avelar, Kristina Strother‐Garcia, Laura M. Justice

**Affiliations:** ^1^ Crane Center for Early Childhood Research & Policy The Ohio State University Columbus Ohio USA

**Keywords:** growth trajectories, late talkers (LT), low income, MacArthur‐Bates Communicative Developmental Inventories (MB‐CDI), vocabulary

## Abstract

Studies show children in low‐income households have heightened risk of developing as late talkers (LTs). Scholars have attributed the cause of these differences to variability in child‐directed and observed language input, parenting quality, attendance at childcare facilities, or some combination therein, as briefly reviewed. However, this study focuses on a sample entirely of families experiencing low income to explore differences within this group. This study explores growth trajectories for child vocabulary production from age 8–30 months in a racially‐diverse low‐income longitudinal sample in the U.S. (*n* = 199). Using multi‐level multiple group models, we explore differences in growth trajectories for LTs and non‐LT peers (identified: age 22–30 months) and identify the age at which vocabulary sizes begin to significantly differ, controlling for the effects of child age‐at‐test, sex, primary home language, and mother's education. Results show distinctly different trajectories, such that: (1) LTs experience relatively flat growth resulting in significantly smaller vocabulary sizes over time and (2) divergence occurs at ∼11 months. Future research is needed to fully understand how and why LT trajectories begin to differ so significantly at this age, and how we can better intervene earlier to reduce the likelihood of LT.

## 1 Introduction

*Late talkers* (LTs) are children exhibiting late language emergence, or “a delay in language onset with no other diagnosed disabilities or developmental delays in other cognitive or motor domains” (American Speech‐Language‐Hearing Association 2024a). LTs are identified using measures of expressive vocabulary size when they are aged 18–35 months (for review, see: Hawa and Spanoudis 2014). LTs are at risk for developing long‐term difficulties in language, literacy, academic, socioemotional, and professional skills (Capone Singleton 2018; Dubois et al. 2020). Previous research suggests that young children residing in homes experiencing low income may be more likely to be identified as LTs (e.g., Avelar et al. 2025; Berger et al. 2009; Hoff‐Ginsberg 1998; Pungello et al. 2009; Singletary et al. 2025). There is an established correlation between socioeconomic status (SES) and child vocabulary size, such that children in households experiencing low income generally have smaller expressive vocabularies than those of children in middle‐ and high‐SES households (e.g., Arriaga et al. 1998; Fisher 2017; Hoff 2003, 2013; Horwitz et al. 2003; Pace et al. 2017). However, families experiencing low income differ substantially in terms of their experiences with economic pressure and hardship (e.g., Justice et al. 2025), such that each family system varies quite a bit, as will the experiences and developmental outcomes of children growing up in each home.

Some scholars suggest that variations in the quantity of child‐directed speech are one of the ways in which experiences differ for children growing up in low‐income households (e.g., Hart and Risely 1995 via the 30‐million‐word gap; Shneidman et al. 2013; Vernon‐Feagans et al. 2020). However, approaches that focus expressly on the quantity of child‐directed input have more recently been viewed as overly simplistic and deficit‐based, and a more holistic approach to embracing the variability and heterogenous nature of within‐SES differences has been suggested (e.g., Miller et al. 2024; Purpura 2019; Raz and Beatty 2018). Family systems with young children, regardless of socioeconomic status, may more importantly differ in terms of the *quality* of child‐directed speech (e.g., Hsu et al. 2017; Rowe and Snow 2020): the verbal environment (which includes overheard speech; e.g., Sperry et al. 2019a, 2019b), parenting quality (which includes caregiver sensitivity and responsivity; e.g., Madigan et al. 2019; Pungello et al. 2009), or some combination therein (e.g., Anderson et al. 2021; Golinkoff et al. 2019; Hoff and Naigles 2002; Rowe 2012). The causes and correlations of these differences in variability in both quantity and quality require deeper investigation both within and across socioeconomic samples to better understand the pathways between parent input and child outcomes.

This paper focuses specifically on late talking in a population of entirely children in low‐income households and seeks to describe how early vocabulary trajectories differ between children who are later identified as LTs as compared to their SES‐matched peers. There is a lack of fine‐grained data on vocabulary growth of LT children prior to the 16‐to‐24‐month age range, around which time they are typically identified as LT (e.g., Hawa and Spanoudis 2014), and particularly there is an underrepresentation of children in low‐income households in prior research, even in epidemiological and cohort studies focused on language delays (e.g., Collisson et al. 2016; Horwitz et al. 2003; Zubrick et al. 2007). The current study aims to address this gap in the literature by analyzing longitudinal vocabulary data from LT children and their non‐LT peers aged 8–30 months from a large, racially diverse sample of American children reared in households experiencing low income.

### 1.1 Late Talkers

The LT designation is generally operationalized using the following cut‐offs regarding a child's expressive vocabulary size at around 24 months of age (Capone Singleton 2018): (a) producing less than 50 words; (b) achieving less than 10th percentile on the MacArthur‐Bates Communicative Developmental Inventories (CDI; Fenson et al. 1993; Marchman et al. 2023); (c) achieving less than 15th percentile on the Language Development Survey (LDS; Rescorla 1989; Rescorla and Alley 2001); or (d) not combining words. The prevalence of LTs is estimated to be 10%–20% in children around 24 months of age (American Speech‐Language‐Hearing Association 2024a). As a group, LTs vary greatly from each other in terms of their individual socio‐demographic characteristics, and as such the previous literature suggests a variety of possible predictors related to a child's risk of delayed language trajectories (e.g., Hentges et al. 2019; Pace et al. 2017; Rescorla 2011). For example, in terms of child‐level characteristics, boys are significantly more likely than girls to meet LT criteria (e.g., Collisson et al. 2016; Hammer et al. 2017; Hentges et al. 2019; Reilly et al. 2007; Zubrick et al. 2007), as are children born early or with low birthweight (e.g., Hammer et al. 2017; Hentges et al. 2019; Zubrick et al. 2007). Children with LT may also exhibit differences in gesture use and language comprehension (e.g., Verganti et al. 2024), as well as in their use of statistical patterns in language learning, which differs substantially from their age‐matched peers (e.g., Simmons and Paul 2024). Additionally, at the level of the parent, a family history of LT (e.g., Collisson et al. 2016; Hentges et al. 2019; Reilly et al. 2007; Zubrick et al. 2007) and lower maternal education (e.g., Hentges et al. 2019; Horwitz et al. 2003; Reilly et al. 2007) are associated with increased LT risk. Finally, at the household level, growing up in a home with more household members (e.g., Giesbrecht et al. 2024; Zubrick et al. 2007), routinely hearing more than one language at home (e.g., Horwitz et al. 2003; Reilly et al. 2007), and being exposed to more screen time (e.g., Giesbrecht et al. 2024; Madigan et al. 2020) are associated with increased LT risk. Other characteristics are associated with decreased LT risk, for example attending childcare centers outside the home and exposure to higher parenting quality inside the home (including informal play, daily book reading, and/or increased sensitivity and responsivity) (e.g., Collisson et al. 2016; Giesbrecht et al. 2024; Hammer et al. 2017). However, the discussion of predictors of LT status, particularly within households experiencing low income specifically, requires more in‐depth study to more fully parse out.

#### 1.1.1 Vocabulary Growth in Late Talkers

There is an abundance of published research on vocabulary acquisition in typically developing children from milestones marking first words onward; however, similar fine‐grained data regarding the early vocabulary development trajectories of LTs, especially prior to 24 months of age, are less common (Hawa and Spanoudis 2014). Rice et al. (2008) reported that LT children followed delayed but otherwise similar vocabulary growth trajectories to two groups of control children, one matched for age and the other matched for baseline mean length of utterance, from age 3–9 years. However, less is known about the earliest stages of vocabulary growth in LTs when they are younger than 36 months of age. In a sample of 28 LTs, Rescorla et al. (2000) used the Language Development Survey (LDS—a checklist of 310 commonly used words) to find that LTs had an average vocabulary size of 18 words at 24 months, 89 words at 30 months, and 195 words at 36 months, showing a stark contrast between LT vocabulary size and the expected average of 150–180 words at 24 months reported across multiple representative community samples totaling over 700 children (reported in Rescorla 1989; Rescorla et al. 1993). Given the low number of LTs in this study, however, follow‐up research is clearly needed to examine LT vocabulary acquisition more closely. Moreover, there is a need to determine whether the vocabulary growth trajectories of LTs diverge from those of non‐LTs before 24 months of age, which could allow for earlier identification and intervention. While there is one recent study by Hentges et al. (2019) that explores the trajectories of language delayed children from 12 to 36 months in a large sample of Canadian children (*N* = 2192), this study uses the Ages and Stages Communication sub‐scale, which does not provide a measure of expressive vocabulary as the measurement across timepoints (12, 24, and 36 months) and only measures expressive vocabulary at 36 months using the CDI. Additionally, participants were predominantly white, middle‐to high‐income, with high levels of maternal education.

There is somewhat more research available on qualitative differences in the compositions of early vocabularies. For example, Perry et al. (2022) found that LTs produced proportionally fewer shape‐based nouns (i.e., words naming solid objects in categories organized by shape similarity, such as *ball* or *cup*) at 13–27 months compared to peers. Researchers have also shown that LTs produce proportionally fewer manner verbs (i.e., verbs that describe the way an action is performed, such as *kick* or *climb*) at 16–30 months compared to peers (Horvath et al. 2022). MacRoy‐Higgins et al. (2016) compared the vocabularies of 12 LTs to those of two control groups, one matched for age and the other matched for total vocabulary size; they found that some differences in vocabulary composition could be explained by phonological characteristics, such that LTs' vocabularies included a greater proportion of phonologically simple words (e.g., animal sounds and sound effects), whereas non‐LTs’ vocabularies were dominated instead by more varied and phonologically complex words (i.e., words for actions, food and drink, and animals). Moreover, Beckage et al. (2011) found that the semantic networks of LTs had less connections than those of non‐LT peers; that is, LTs' vocabularies were made up of words that were more distally related to each other in meaning. These differences in vocabulary composition likely result from differences in word‐learning biases (Jones 2003; Perry et al. 2022), phonological processing (Carson et al. 2003; MacRoy‐Higgins et al. 2016), and semantic organization (Beckage et al. 2011).

#### 1.1.2 Long‐Term Outcomes of Late Talking

The majority (about 50%–70%) of LTs go on to perform within normal limits on language assessments at a later timepoint (Dale et al. 2003; Paul and Weismer 2013), leading many to refer to this subset of LTs as *late bloomers*. Late bloomers are often said to have “caught up” to their typically developing peers, but research shows that late bloomers are susceptible for experiencing persistent sub‐clinical language and reading difficulties throughout childhood and into adolescence (Fisher 2017; Paul and Weismer 2013; Rescorla 2011). For instance, children with a history of being LTs had lower scores in oral narrative skills, syntax, and story grammar at ages 8–9 years compared to their peers with no history of late talking (Manhardt and Rescorla 2002). Furthermore, even though LTs scored in the average range in language and reading assessments at age 13 years (Rescorla 2005) and 17 years (Rescorla 2009), they had weaker vocabulary, grammar, and verbal memory scores than their non‐LT peers at both ages. Despite these scores not being extreme enough to warrant a diagnosis of language or reading disorder, they may nonetheless have a negative impact on children's cognitive, social, and academic development (Hammer et al. 2017; Rescorla 2011). For example, late bloomers score lower on measures of language, reading, social skills, and emotional regulation compared to typically developing peers (Capone Singleton 2018). When language delays persist into the third year of life, the chances of recovering and catching up to peers is reduced (Paul 1993).

LTs who do not end up in the late bloomer category are likely to meet criteria for developmental language disorder (DLD), as they do not catch up to their peers in the same way that late bloomers do. DLD is a spoken language disorder that constitutes “a primary disability without a known medical cause and persisting at school age and beyond” or that “co‐occurs with other diagnoses such as attention‐deficit/hyperactivity disorder or developmental coordination disorder” without an obvious causal relationship (American Speech‐Language‐Hearing Association 2024b). While many researchers and clinicians now use the term DLD in place of specific language impairment (SLI), it should be noted that some continue to use SLI to refer to this condition, and others define SLI using somewhat stricter criteria that distinguish it from DLD (e.g., Rice 2020; for further discussion of terminology, see Bishop et al. 2017). DLD is a lifelong condition that, though treatable, has been associated with a variety of academic, professional, and socioemotional challenges, including lower rates of school completion, full‐time employment, and self‐confidence (for a review, see Dubois et al. 2020). Although not all LTs will go on to be diagnosed with DLD, and not all individuals with DLD are former LTs (Poll and Miller 2013), there is a significant need to improve our understanding of the emergence of LT as a potential route for improving early identification and, when necessary, intervention.

### 1.2 The Current Study

Several studies have found correlations between income and child vocabulary size in the U.S. (e.g., Berger et al. 2009; Hoff‐Ginsberg 1998; Pungello et al. 2009), suggesting that young children residing in homes experiencing low income may be more likely to be identified as LTs; however, no large‐scale study published to date has reported specifically on the relative prevalence of LTs among American children in low‐income households (for reviews of the literature, see: Fisher 2017; Hawa and Spanoudis 2014; Rescorla 2011). Studies from other Western contexts are suggestive of increased LT prevalence for children in low‐income households, however. In a large sample (*N* = 712) of British children from households experiencing low income, the majority of whom belonged to minority ethnic groups (90%) and were bilingual (82%), Cheung et al. (2023) found the incidence of LTs to be 24.86%, significantly higher than rates reported in previous studies of monolingual English‐speaking children who were mostly White and came from mainly middle‐ and high‐SES households (9.6%–19.1%; e.g., Armstrong et al. 2017; Collisson et al. 2016; Dale et al. 2003; Reilly et al. 2007; Rescorla et al. 1993; Zubrick et al. 2007). Coupled with existing research on SES‐related vocabulary development gaps (e.g., Berger et al. 2009; Hoff‐Ginsberg 1998; Pungello et al. 2009), this suggests that LT rates could differ among American children from different sociodemographic backgrounds. However, the only large‐scale studies of LTs in diverse samples published in peer‐reviewed journals in the last 25 years drew upon data from outside the U.S. (e.g., Armstrong et al. 2017; Cheung et al. 2023; Collisson et al. 2016; Dale et al. 2003; Henrichs et al. 2011; Reilly et al. 2007, 2011; Rice et al. 2008; Rice et al. 2014; Roulstone et al. 2002; Schjølberg et al. 2011; Westerlund et al. 2006; Zubrick et al. 2007). Large‐scale studies in the U.S. have included samples that were not racially and socioeconomically diverse (e.g., Hammer et al. 2017; Horwitz et al. 2003; Morgan et al. 2020) or samples with unspecified sociodemographic characteristics (e.g., Horvath et al. 2022; Perry et al. 2022; Poll and Miller 2013; Preston et al. 2010).

Notably, families experiencing low income in the U.S. face unique difficulties compared to those in other countries which provide universal health care, guaranteed paid parental leave, and generally have lower rates of child poverty than the U.S. (e.g., Australia, the U.K. and Canada). Low‐income parents in the U.S. often struggle to access support services for financial and developmental needs associated with raising a child, as well as quality medical care, childcare, housing, and social support, which can impact their parenting behaviors (Cook et al. 2024). Additionally, systemic barriers like racism and historical trauma can also affect parenting in families experiencing low income (e.g., Sege et al. 2022). As one of the proposed mechanisms linking increased risk of LT in children to experiences of low income is the quality of parent‐child interactions (e.g., Hawa and Spanoudis 2014; Hirsh‐Pasek et al. 2015; Hoff 2003; Justice et al. 2019; Rowe 2008), these unique difficulties that U.S. families face could play a role in influencing language development through their impact on parental stress and parenting quality creating differences in growth trajectories compared to what we see in other countries.

Importantly, researchers have documented substantial variation in child and parent language use *within* SES groups (Hoff 2013; Schwab and Lew‐Williams 2016), indicating that children from households experiencing low income are not a homogenous group with respect to vocabulary development. Nonetheless, SES‐related achievement gaps in language and literacy persist (Golinkoff et al. 2019), and according to the U.S. Census Bureau's supplemental poverty measure, the poverty rate among American children more than doubled from 5.2% in 2021 to 12.4% in 2022 (for details, see Shrider and Creamer 2023). A clearer picture of vocabulary growth trajectories among LTs and their typically developing peers among American children in low‐income households would be informative for best clinical and educational practice, including whether and when to intervene to potentially address late‐language emergence in affected children. This need has only become more critical since the so‐called “wait‐and‐see” approach to identify LTs is no longer recommended by expert clinicians, due to the long‐term risks associated with delayed language acquisition (Capone Singleton 2018).

Given the likelihood of a higher prevalence of LTs in low‐income populations and the general lack of large longitudinal datasets documenting early language growth trajectories in these populations in the U.S., the current study fills a gap in the literature by analyzing longitudinal data collected entirely from families experiencing low income. The current study uses a sample of families living in Ohio—a state with a U.S. Census Bureau 3‐year average supplemental poverty measure of 7.3% from 2020 to 2022 (ranked 33rd of 50 states) compared to the national U.S. 3‐year average of 9.8% for the same period (Shrider and Creamer 2023), suggesting that Ohio families during this period experienced slightly less poverty compared to the nation as a whole, but were relatively similar. The current study is the first to examine the trajectories of vocabulary growth in both LTs and their non‐LT peers longitudinally from 8 to 30 months of age thereby allowing an examination of how these trajectories may differ and if so, when this divergence occurs. Obtaining a better understanding of the growth trajectories of vocabulary acquisition of LTs is important to develop early interventions and provide better support for children and their parents as well as to further understand the mechanisms that may affect the differences in their growth trajectories. The current study addressed two aims: (1) to examine vocabulary size growth over time to determine whether LTs show a *delayed* growth trajectory or a *different* growth trajectory, compared to non‐LT peers; and (2) to identify the age at which LT and non‐LT growth trajectories diverge. We use multi‐level multiple group models to examine growth trajectories in vocabulary size for LTs and their non‐LT peers and to determine the timing at which this divergence in vocabulary size occurs. Children were assessed once for LT status, when they were between 22 and 30 months of age (or ∼2 years old), and this status was then used to understand growth trajectories from three additional timepoints preceding this assessment (when children were between 8 and 25 months of age, as described below).

## 2 Methods

### 2.1 Study Participants

Participants for the current study include a sub‐sample of participating dyads from the longitudinal study *SMALL Talk: A Study of Milestones to Advance Language Learning* (*N* = 356 parent‐child dyads living in the midwestern United States). As the primary aim of SMALL Talk is to better understand the high risk and prevalence of LT children growing up in low‐income homes, our sample consists entirely of dyads experiencing low income. Prior to the onset of the COVID‐19 Pandemic in March 2020, our team attended local events and centers serving low‐income families in central Ohio to recruit participants (*n* = 20 dyads). However, after March 2020, to continue to reach potential participants from low‐income homes during the pandemic, we asked local Women, Infants, Children (WIC) centers to send text advertisements to their eligible families (*n* = 336 dyads). SMALL Talk dyads were initially recruited between October 2019 and December 2020 (*n* = 337). Likely because individuals were facing unprecedented stress and upheaval to their normal routines following the onset of the COVID‐19 Pandemic, we experienced attrition following the first timepoint of data collection (*n* = 18 withdrawals), such that between August and November 2021 we recruited additional dyads during a replenishment period to account for early attrition to the original sample (*n* = 19, joined the study at the second timepoint).

Prior to consenting to enroll, participants were screened by an online survey. To address the primary inclusionary criterion (i.e., living in a home experiencing low income), we first asked if dyads were currently receiving government services related to income (e.g., WIC, food stamps, CareSource, Medicaid, or housing subsidies). If a potential participant indicated that the dyad did not currently receive services, we used their annual household income and household size to calculate the dyad's relation to the federal poverty level, such that those at less than 200% of the 2019 federal poverty level qualified. Additional inclusionary criteria for adult participants included: being ≥ 18 years of age and having a child between 6 and 15 months old at the time of enrollment; being comfortable reading and speaking in English; and having no immediate plans to move out of the general area. While the final SMALL Talk sample was composed primarily of biological mothers (*n* = 355, or 99.7% of the sample), primary caregivers were allowed to enroll if they had legal custody of the child (however, only one biological grandmother with legal custody of the child enrolled). Thus, henceforth adult participants are referred to as *mothers*. Finally, inclusionary criteria for children included having been born from a singleton birth at ≥ 35‐weeks’ gestation, with no diagnoses of significant disabilities at or around the time of birth. The present study was conducted according to guidelines laid down in the Declaration of Helsinki, with written informed consent obtained from a parent or guardian (for their own participation and for each child) before any assessment or data collection occurred. This study was approved by The Ohio State University's Institutional Review Board (IRB Protocol: 2019B0220—SMALL Talk: Study of Milestones to Advance Language Learning).

In terms of our analytical sample for the current study, 199 mother‐child dyads were included (see Table 1 for demographics). Mothers identified predominantly as non‐Hispanic (92%), and reported their race as follows: Black (42%), White (42%), and Other (17%). Most families spoke English as their primary language in the home (86%), although other primary languages included (but were not limited to): Spanish, Igbo, Nepali, Twi, and Arabic. Approximately half (55.8%) of the children's mothers obtained a high school diploma or GED as their highest degree earned, 30.2% held a college degree, and 14.1% had less than a high school degree. On average, families reported their annual income at around $27,000, with about 4.35 people living at home. At the time of the final data collection used in this study (children = ∼25 months of age), 35% of children were enrolled in daycare outside the home.

**TABLE 1:** Sociodemographic characteristics of identified LTs versus non‐LT peers in the analytical sample.

	Late talkers (TP4, *n* = 58, 29%)		Peers (TP4, *n* = 141, 71%)		*p*‐value^a^
	Mean or %	SD	Mean or %	SD	
Child age‐at‐test in months					
TP1 (*n =* 123)	8.48	1.84	8.48	1.54	0.988
TP2 (*n =* 166)	14.37	1.20	14.26	1.07	0.541
TP3 (*n =* 184)	19.84	1.40	19.95	1.21	0.617
TP4 (*n =* 199)	25.16	1.79	25.50	1.90	0.222
Child sex: Girl	43%		47%		0.634
Child ethnicity: Hispanic	14%		6%		0.056
Child's race					**0.029**
Black	38%		43%		
White	34%		44%		
Other (including multiracial)	29%		13%		
Primary home language: English	81%		89%		0.154
Maternal education					0.733
Less than high school degree	17%		13%		
High school degree/GED	50%		58%		
College degree or higher	33%		29%		
Annual household income					0.533
$10,000 or less	30%		23%		
$10,001–$20,000	17%		14%		
$20,001–$30,000	26%		23%		
$30,001–$40,000	15%		20%		
$40,001 or more	13%		20%		

*Note:* Bolded values indicate significant differences between LTs and their non‐LT peers (*p* < 0.05).

^a^ We conducted ANOVA to examine differences between profiles for continuous variables, and Chi‐squared tests to examine differences between profiles for categorical variables. For continuous variables, mean values and standard deviations were reported. For categorical variables, the percentage of each category was reported.

For the current study, we used vocabulary measures collected from our analytical sample across four time‐points, totaling 672 scores. With the sample sizes of 672 at level‐1 (i.e., the longitudinal observation level) and of 199 at level‐2 (i.e., the participant level), we conducted a priori power analyses of two‐level models with various levels of intra‐class correlation (ICC = 0.1, 0.2, 0.3, 0.4, 0.5), assuming that covariates account for 20% of the variance at each level. With power of 0.8, the minimum detectable effect sizes ranged from 0.11 to 0.14 at level‐2, and 0.07 to 0.09 at level‐1. Therefore, we are sufficiently‐powered to detect small effect sizes.

### 2.2 Study Procedure

Throughout the longitudinal SMALL Talk study, participants were asked to complete timepoints about two to three times per year, depending on the child's age and dyad's availability (or about every 3–6 months). For the first four timepoints of SMALL Talk (i.e., the period of interest for the current study), each timepoint consisted of a scheduled interview‐based phone call with trained staff (∼30–60 min to complete), followed by emailed surveys completed at the mother's convenience in the following weeks (additional ∼30–60 min to complete). Notably, age‐at‐test variables were calculated for all child outcome variables such that the age‐at‐test = the recorded date for the data—the child's birthdate; as such, differences in when surveys were completed by mothers were always accounted for by inclusion of age‐at‐test as a covariate in resulting datasets.

Mothers received gift cards for completing each timepoint, such that they received $35 for each timepoint if they completed all phone and emailed surveys. Participants also received an extra $10 *thank you* gift card for completing the second timepoint, denoting having stayed in the study for the first year of the project. Notably, participants were able to skip any portion of any timepoint and still participate in the study at the next timepoint, resulting in varying sample sizes for each measure at each timepoint throughout the study—failure to participate in any given timepoint could be attributable to time involvement, study fatigue, life stress encroachment, personal preference, or some combination of these things (participants were not asked to provide reasons for skipping any study portions or timepoints).

### 2.3 Measures

To assess vocabulary growth over time for LTs and their non‐LT peers, we used: (1) child productive vocabulary collected at each of the first four timepoints of SMALL Talk, (2) LT status as identified when children were 22–30 months of age (at the fourth timepoint, hereafter TP4), and (3) key sociodemographic predictors including child sex, home language, and maternal education (collected at the time of enrollment) and child age‐at‐test (calculated using the child's birthdate and the completion date of the vocabulary measure). Because LT status was the primary child characteristic of interest in the current study, the analytical sample for this paper was comprised of *n* = 199 mother‐child dyads who provided data at TP4, while individuals from the SMALL Talk study who were missing data at this timepoint were excluded. Thus, throughout the rest of the paper, sample sizes denoted refer to data relevant to the analytical sample specifically.

Data for the first timepoint (hereafter TP1) were collected between January 2020 and March 2021 during the initial enrollment period, when children were 9.2 ± 1.6 months old (range 8–14 months; *n* = 123), and thus did not include replenishment dyads. Data for the second timepoint (hereafter TP2) were collected between August 2020 and November 2021 and included replenishment dyads (at their time of enrollment), when children were 14.2 ± 0.9 months old (range 11–17 months; *n* = 166). Data for the third timepoint (hereafter TP3) were collected between January 2021 and May 2022, when children were 19.9 ± 1.2 months old (range 18–25 months; *n* = 184). Finally, data for TP4 were collected between June 2021 and December 2022, when children were 25.4 ± 1.9 months old (range 22–30 months; *n* = 199).

As noted above, scheduling of each timepoint was driven by the mother's availability, such that timepoints occurred approximately 3–6 months apart and children were as *close as possible* to the target age for that timepoint. As such, age‐at‐test was calculated for every child outcome variable, as not every child fell within the targeted age range at the time the mother was available for data collection. Additionally, when necessary, if the child's age‐at‐test fell outside the range acceptable for norm‐referenced outcomes, they did not receive scores for those variables and instead received missing data values.

#### 2.3.1 Child Productive Vocabulary

To measure child productive vocabulary, we used the Words Produced sub‐scale of the Communicative Development Inventories (CDI; Fenson et al. 1993, 2007; Marchman et al. 2023). We used the Words and Gestures form (CDI‐WG), when children were aged 8–17 months at TP1 and TP2, and the Words and Sentences form (CDI‐WS), when children were aged 18–30 months at TP3 and TP4. The Words Produced sub‐scale for the CDI‐WG includes a list of 396 words, while the CDI‐WS includes a list of 680 words. At each timepoint, the mother was asked to indicate *Yes* = 1 or *No* = 0 regarding whether the child could say each listed vocabulary word in English. However, mothers were also provided options for *I don't know* or *Prefer not to answer*, which resulted in the potential for some missing data. At each timepoint, the child received a Words Produced sum score representing their total productive vocabulary. However, because of the possibility of missing data, a sum score was only calculated for participants with < 20% total missing data across all vocabulary words (i.e., for those who answered at least 317 items on the CDI‐WG at TP1/TP2, or at least 544 items on the CDI‐WS at TP3/TP4). Notably, if children were outside the validated age range of the assessment at the time of their mother completing the corresponding timepoint (i.e., under 8 months or over 18 months at TP1 or TP2, or under 16 months or over 30 months at TP3 or TP4), these individuals received missing data values for their CDI score.

#### 2.3.2 Late Talker Status

We also use the CDI Words Produced sum score to identify LT status, as it is common practice to use productive vocabulary as a measure for identifying LTs (e.g., Bleses and Vach 2013; Collisson et al. 2016; Desmarais et al. 2008; Horwitz et al. 2003; Korpilahti et al. 2016; Rescorla 1989). At TP4, when the participating children were 22–30 months of age (mean = 25.40, SD = 1.87), the CDI‐WS Words Produced sum score was converted to a percentile rank score for each child, referencing the CDI third edition normative sample adjusted by child sex and age (Marchman et al. 2023). Subsequently, children scoring at or below the 10^th^ percentile were identified as LTs, while children scoring above the 10^th^ percentile were identified as non‐LTs, hereafter referred to as *peers* (e.g., Avelar et al. 2025; Bleses and Vach 2013; Collisson et al. 2016; Desmarais et al. 2008; Horwitz et al. 2003; Korpilahti et al. 2016; Rescorla 1989).

#### 2.3.3 Sociodemographic Covariates

We included the following sociodemographic variables in our analyses: child age‐at‐test, child's sex, primary home language, and maternal education, as each of these variables has been previously shown to correlate with early child language outcomes (e.g., Buac et al. 2014; Fenson et al. 1994; Huttenlocher et al. 1991; Justice et al. 2020; Quiroz et al. 2010). Child age‐at‐test (in months) was calculated for each timepoint based on the timestamp of completion for the corresponding CDI responses. The remaining covariates were each collected at the time of enrollment. Child's sex was coded as *Male* = 0 or *Female* = 1. The primary home language was coded as a dichotomous variable for analyses as *English* = 0 or *Another language* = 1. Maternal education was coded as: *Less than high school degree* = 1, *High school degree/GED* = 2, *College degree or higher* = 3, based on the highest level of degree achieved. Within the analyses, *Less than high school degree* was used as the reference group. Table 1 displays demographic characteristics of the subsamples of typically developing children as well as children identified as LTs.

Note that children from households whose primary language was not English (14% of the analytical sample) were also identified with the same criteria, and 41% of them scored below the 10th percentile threshold when measured in English vocabulary, as compared to 27% of the children from English‐speaking households who scored below the threshold. Notably, the prevalence of LTs in our sample was relatively high, regardless of whether we included children whose primary home language was English or any other language (*n* = 199; 29% or 58 LTs vs. 141 non‐LTs), or if we only considered children with English as their primary language (*n* = 172; 27% or 47 LTs vs. 125 non‐LTs). Since the correlation between home language and LT status was relatively low (*r* = 0.10, *p* > 0.05), we decided to include children whose primary language was not English in our analytical sample. We further tested all models including or excluding the sample of children whose primary language was not English to verify that the results did not substantially differ based on primary language exposure, and found results were robust and consistent in both cases, suggesting that the results are not an artifact of the inclusion of English‐language learners (see Table S1 for results including only English primary language children). Thus, retaining children whose primary language was not English allowed us to have a larger more representative sample, especially since the outcomes were nearly identical with and without the inclusion of non‐English primary language children. While inclusion of information about the percentage of time that children were exposed to English and non‐English languages in their homes may be impactful, we did not collect data about this and thus cannot further parse out how primary home language exposure impacts vocabulary development in LTs and their peers.

### 2.4 Analytic Strategy

We used CDI Words Produced sum scores longitudinally across four timepoints to represent children's language growth trajectories for LTs versus peers. Because CDI‐WG was administered at TP1 and TP2, while the CDI‐WS was administered in the subsequent two timepoints, we converted CDI Words Produced sum scores obtained from the two forms of the assessment to the same scale of converted *vocabulary size estimate* based on the formula provided by Mayor and Plunkett (2010), (2011), but see Table S2 for descriptives of scale scores prior to conversion (i.e., for comparison to other study populations). Mayor and Plunkett (2011) found that CDI Words Produced sum scores presented systematic underestimation of children's vocabulary size, and the discrepancy increased dramatically with age. Therefore, they linked CDI‐WG and CDI‐WS Words Produced sum scores to total vocabulary size estimate based on item response models, which correct for common words as well as idiosyncratic individual words absent from the CDI. Due to the extreme skewed distribution of the vocabulary size estimate, we then log‐transformed these variables before including them in our analyses. To ensure that all transformed values are valid numbers and that a vocabulary size estimate of zero corresponds to the zero point on the log‐transformed scale, we applied the formula: Transformed vocabulary size = ln(Vocabulary size estimate + 1).

A two‐level multiple‐group linear model was used to characterize the growth trajectories in log transformed vocabulary size estimates for LTs (*n* = 58) versus peers (*n* = 141). Measures of vocabulary size estimates of up to four timepoints per child at level‐1 (*n* = 672) were predicted by age‐at‐test and nested within individuals at level‐2 (*n* = 199) where covariates included child‐level attributes. Tables 1 and 2 list the variables used in the analyses and our sample descriptive statistics. The multilevel multigroup model allows for evaluation and comparison of each group's growth parameters while accounting for the nested structure of the data. Specifically, three models were tested by adjusting constraints in different model components. The first model (Invariance Trajectory Model) assumes that the two groups have the same growth trajectories, including intercept, slope, and error variances. The second model (Free Slope Model) assumes that the two groups may have different slope in vocabulary growth. The third model (Free Intercept and Slope Model) assumes that the two groups may have completely different growth trajectories, including intercept and slope. By relaxing the restrictions and comparing model fit, we aim to find the best fitting model that describes growth characteristics of LTs and peers. Model fit statistics including Akaike Information Criteria (AIC), Bayesian Information Criteria (BIC), and Chi‐square difference tests were used to compare the models. In this process, we also estimated slopes (i.e., change rate) of log transformed estimated vocabulary growth for children who were LTs and peers and compared the differences between these parameters.

**TABLE 2:** Descriptive statistics of variables for multilevel multiple‐group growth model.

Variables	Description
Outcome	
Log‐transformed vocabulary size^a^	Mean = 3.70, SD = 2.11, range = 0.00–8.04
Level 1	
Child age‐at‐test in months	Mean = 18.17, SD = 6.07, range = 8–30
Level 2	
Sex	Male = 54%
	Female = 46%
Primary home language	English = 86%
	Another language = 14%
Maternal education^b^	Less than high school degree = 14.1%
	High school degree/GED = 55.8%
	College degree or higher = 30.2%

^a^ Vocabulary size estimates are based on the conversion formula (Mayor and Plunkett 2010, 2011) used to convert CDI‐Words and Gestures and CDI‐Words and Sentences Words Produced sum scores to the same scale producing a *converted vocabulary size estimate.* Due to the extremely skewed distribution of the vocabulary size we applied log transformation: log(vocabulary size estimate + 1).

^b^ The model does not compare high school degree/GED versus College degree or higher, because less than high school degree was used as the reference group.

We further conducted a series of tests to pinpoint the age at which LTs and peers started to significantly diverge in their vocabulary size estimates. We changed the centering point of the level‐1 predictor, child age‐at‐test (from 8 to 30 months), and compared the intercepts of the two groups, such that the age(s) at which intercepts significantly differed represented that at which the two groups of children had significantly different vocabulary size estimates. We also estimated the effect size of the group differences.

All multi‐level multiple group models were conducted using Mplus (Muthén and Muthén 1998–2017). In our analytical sample, as defined by having data for LT evaluation from TP4 (*n* = 199), missing data ranged from 0% to 38% for CDI and corresponding age‐at‐test data (TP1, CDI‐WG: 38%, TP2, CDI‐WG: 17%, TP3, CDI‐WS: 8%, TP4, CDI‐WS: 0%). There was no missing data in other covariates. Multilevel models can naturally accommodate unequal number of measurement occasions for different individuals, and thus we used full maximum likelihood (FIML) estimation method to treat all missing data. FIML assumes that data are missing at random (MAR). It is usually reasonable to assume MAR unless there are theoretical reasons to argue otherwise, and techniques such as FIML are robust to mild deviation from MAR (Collins et al. 2001; Schafer and Graham 2002).

## 3 Results

### 3.1 Description of the Analytical Sample

The analytical sample for the current study consisted of the 199 children evaluated for LT status at TP4, when the children were on average 25.4 months old. Within this sample, 54% were boys and 46% were girls. Most children came from households that primarily spoke English (86%). At TP4, 29% of the children were identified as LTs, while the rest were identified as non‐LTs. Table 3 provides the vocabulary size estimates for LTs and their peers at each timepoint, based on the formula provided by Mayor and Plunkett (2010, 2011), which represent the data that were used in subsequent analyses before log‐transformation.

**TABLE 3:** Vocabulary size estimates for late talkers and peers at each timepoint, and each month (8–30 months).

	Late talkers (TP4 *n* = 58, 29%)	Peers (TP4 *n* = 141, 71%)	Test of difference (*p*)
Timepoints			
TP1 (*n* = 123)	3.63 (4.89)	5.60 (8.23)	0.102
TP2 (*n* = 166)	9.65 (11.80)	37.77 (107.41)	0.006
TP3 (*n* = 184)	29.56 (37.11)	300.53 (434.32)	< 0.001
TP4 (*n* = 199)	47.53 (42.33)	882.32 (804.52)	< 0.001
Age in months			
8 months	1.54 (2.03)	3.32 (3.45)	0.046
9 months	4.27 (6.71)	3.73 (3.54)	0.804
10 months	3.00 (3.06)	8.16 (11.04)	0.047
11 months	7.20 (5.17)	13.60 (18.15)	0.485
12 months	N/A	10.50 (6.36)	N/A
13 months	9.63 (12.42)	34.50 (35.50)	0.008
14 months	6.64 (8.37)	26.76 (50.31)	0.004
15 months	11.90 (14.03)	62.73 (213.73)	0.239
16 months	5.75 (4.99)	40.90 (31.48)	0.006
17 months	29.00 (18.39)	N/A	N/A
18 months	N/A	81.33 (51.16)	N/A
19 months	25.22 (42.16)	191.80 (287.34)	< 0.001
20 months	24.33 (17.22)	333.86 (312.02)	< 0.001
21 months	33.75 (16.62)	566.50 (837.94)	0.023
22 months	32.00 (27.82)	212.56 (259.83)	0.072
23 months	43.33 (39.38)	476.69 (456.72)	0.002
24 months	31.35 (31.57)	825.00 (841.04)	< 0.001
25 months	70.77 (43.90)	712.97 (696.19)	< 0.001
26 months	81.22 (58.61)	594.32 (341.92)	< 0.001
27 months	35.33 (29.54)	1970.00 (1035.31)	0.001
28 months	39.50 (30.41)	1389.27 (885.85)	< 0.001
29 months	29.67 (9.24)	1526.11 (845.79)	< 0.001
30 months	N/A	787.33 (350.71)	N/A

*Note:* Vocabulary size estimates are based on a conversion formula (Mayor and Plunkett 2010, 2011) used to convert CDI‐Words and Gestures and CDI‐Words and Sentences Words Produced sum scores to the same scale producing a *converted vocabulary size estimate*, represented here. Mean values of vocabulary counts are shown for each TP and age, and standard deviations are shown in parentheses; values were calculated based on available data so each month's statistics are based on a different subsample depending on when each child was assessed. N/A means that not enough data are available to calculate the summary statistics.

### 3.2 Multiple Group Analyses

Centering child age at the baseline (8 months), we tested three models for multiple group analyses: first, the “Invariance Trajectory Model,” where intercept, slopes and error variances were assumed to be the same across groups; second, the “Free Slope Model,” where slopes were free to vary but intercepts were not; and, finally, the “Free Slope and Intercept Model,” where both slope and intercept were free to vary. Fit statistics are summarized in Table 4, which shows that the Free Slope Model provides the best fit among the three (*χ* ^2^ = 14.52, *df* = 13, *p* = 0.339; AIC = 7037.00, BIC = 7194.85, SSBIC = 7083.73), and that freeing of intercepts does not significantly improve model fit (*p* = 0.996). Therefore, growth parameters were estimated for LTs and peers based on the Free Slope Model (see Table 5).

**TABLE 4:** Comparison of multiple‐group linear growth models.

Model	*χ* ^2^	*df*	Difference *p*‐value^a^	AIC	BIC	SSBIC
Invariance Trajectory Model	195.50	14		7204.86	7358.21	7250.26
Free Slope Model	14.52	13	< 0.001	7037.00	7194.85	7083.73
Free Slope + Intercept Model	14.51	12	0.996	7039.00	7201.37	7087.06

*Note:* Outcome is the log‐transformed vocabulary size. Intercept of the model was set at 8 months old.

Abbreviations: AIC = Akaike Information Criteria; BIC = Bayesian Information Criteria; SSBIC = Sample‐Size Adjusted BIC.

^a^ *p*‐values are from the Satorra–Bentler scaled chi‐square difference tests (Satorra and Bentler 2010) that examine the difference in chi‐square values after changes in constraints.

**TABLE 5:** Two‐level two‐group linear model (*N* = 199): Log‐transformed vocabulary growth trajectories for late talkers and peers.

	Peers		Late talkers	
	*β* (SE)	*p*	*β* (SE)	*p*
Fixed effects				
Intercept at 8 months^a^	0.87 (0.16)	< 0.001	0.87 (0.16)	< 0.001
Age in months	0.31 (0.01)	< 0.001	0.15 (0.01)	< 0.001
Sex: 1 = female	0.43 (0.14)	0.002	0.08 (0.18)	0.661
Home language: 1 = another language	−0.39 (0.20)	0.051	−0.29 (0.28)	0.304
Maternal education^b^				
High school degree/GED versus no high school degree/GED	0.14 (0.20)	0.477	0.29 (0.24)	0.233
College degree or higher versus no high school degree/GED	0.02 (0.20)	0.907	−0.08 (0.23)	0.736
Random effects				
Level 1	0.63 (0.05)	< 0.001	0.63 (0.05)	< 0.001
Level 2	0.44 (0.06)	< 0.001	0.44 (0.06)	< 0.001

^a^ Free Slope Model was used where the intercept of the growth curve (8 months) was held constant across two groups. Outcome is the log‐transformed vocabulary size. Intercept of the model was set at 8 months old, or the youngest age when CDI was administered. Model fit: *χ* ^2^ = 14.52, *df* = 13, *p* = 0.339. RMSEA = 0.019. CFI = 0.998, TLI = 0.997. SRMR within = 0.011, SRMR between = 0.066.

^b^ The model does not compare high school degree/GED versus College degree or higher, because less than high school degree was used as the reference group. *β* = unstandardized coefficient; SE = standard error.

**FIGURE 1:**
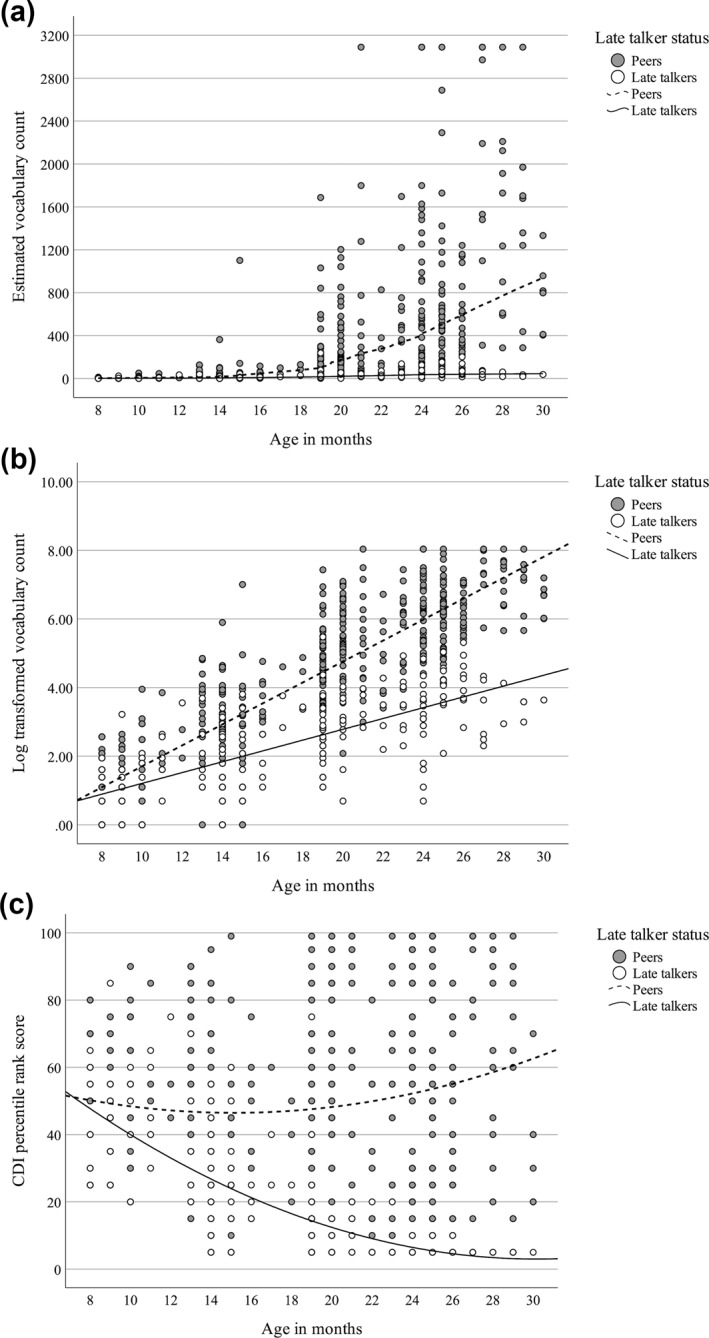
Vocabulary size ([a] estimated; [b] log transformed; [c] percentile rank) from 8 to 30 months: Scatterplots and fit lines. Vocabulary size estimates are based on the conversion formula (Mayor and Plunkett 2010, 2011) used to convert CDI‐Words and Gestures and CDI‐Words and Sentences Words Produced sum scores to the same scale producing a *converted vocabulary size estimate.*

A Wald's test on slope differences further confirmed our hypothesis that LTs and peers grew in vocabulary size estimates at a significantly different rate from 8 to 30 months (*p* < 0.001). Figure 1 displays the scatterplot of estimated and log‐transformed vocabulary counts by age and the fit lines by group. Although both groups showed vocabulary size estimate growth as their ages increased, LTs obtained vocabulary size at a much slower rate than their peers. The log growth rate for peers is 0.31 per month, whereas it is only 0.15 per month for LTs. This translates to exponential differences in estimated vocabulary size growth between the two groups.

### 3.3 Test for Divergence Point

Based on the Free Slope Model, we further explored the timepoint at which LTs and peers started to diverge significantly in vocabulary size estimates by changing the centering point of age as a predictor. We also re‐centered the covariate values to estimate the trajectories for the “average” child in our sample, that is, the ones with average scores on all covariates. Using a sequence of tests between group intercepts, we found that the log transformed vocabulary size estimates for the two groups started to diverge significantly at 10 months (*p* = 0.013, effect size *d* = 0.38; see Table 6). Effect size reached moderate size (*d* > 0.50) at 11 months. We also evaluated the robustness of the divergence point using percentile rank scores from CDI assessments (see Table 6). The models using this alternative measure of outcome yield consistent results, where LTs and peers started to diverge significantly in CDI percentile ranks around 10 months (*p* = 0.006, *d* = 0.41). With the differences in percentile rank scores, effect size reached moderate magnitude at 11 months. These analyses indicate that the gap in vocabulary size between LTs and their peers appeared around 1 year of age.

**TABLE 6:** Differences in log transformed vocabulary size and percentile ranks from 8 to 30 months: Estimates from the multilevel growth models.

	Estimated log‐transformed vocabulary				Estimated percentile rank of vocabulary			
	Late talkers	Peers	Wald test of difference (*p*)	Estimated effect size (*d*)	Late talkers	Peers	Wald test of difference (*p*)	Estimated effect size (*d*)
	*β* (SE)	*β* (SE)			*β* (SE)	*β* (SE)		
Age in months								
8 months	1.00 (0.15)	1.10 (0.10)	0.604	0.09	48.36 (3.09)	51.01 (2.30)	0.493	0.10
9 months	1.16 (0.14)	1.40 (0.10)	0.146	0.21	44.42 (2.72)	49.85 (1.95)	0.105	0.24
10 months	1.31 (0.13)	1.71 (0.09)	0.013	0.38	40.66 (2.41)	48.86 (1.72)	0.006	0.41
11 months	1.46 (0.12)	2.01 (0.09)	< 0.001	0.54	37.07 (2.17)	48.03 (1.60)	< 0.001	0.60
12 months	1.61 (0.12)	2.32 (0.09)	< 0.001	0.69	33.66 (1.99)	47.36 (1.59)	< 0.001	0.77
13 months	1.77 (0.11)	2.62 (0.08)	< 0.001	0.93	30.42 (1.86)	46.86 (1.65)	< 0.001	0.90
14 months	1.92 (0.11)	2.93 (0.08)	< 0.001	1.10	27.36 (1.77)	46.51 (1.74)	< 0.001	1.02
15 months	2.07 (0.11)	3.23 (0.07)	< 0.001	1.39	24.47 (1.70)	46.34 (1.84)	< 0.001	1.11
16 months	2.22 (0.10)	3.54 (0.07)	< 0.001	1.63	21.76 (1.65)	46.32 (1.94)	< 0.001	1.19
17 months	2.37 (0.10)	3.84 (0.07)	< 0.001	1.81	19.22 (1.59)	46.47 (2.01)	< 0.001	1.29
18 months	2.53 (0.10)	4.15 (0.07)	< 0.001	2.00	16.86 (1.52)	46.79 (2.06)	< 0.001	1.39
19 months	2.68 (0.10)	4.45 (0.07)	< 0.001	2.18	14.68 (1.43)	47.26 (2.08)	< 0.001	1.51
20 months	2.83 (0.10)	4.76 (0.07)	< 0.001	2.38	12.66 (1.31)	47.90 (2.09)	< 0.001	1.63
21 months	2.98 (0.11)	5.06 (0.07)	< 0.001	2.50	10.83 (1.17)	48.70 (2.09)	< 0.001	1.76
22 months	3.13 (0.11)	5.37 (0.07)	< 0.001	2.69	9.16 (1.00)	49.66 (2.09)	< 0.001	1.90
23 months	3.29 (0.11)	5.67 (0.07)	< 0.001	2.86	7.68 (0.81)	50.79 (2.11)	< 0.001	2.02
24 months	3.44 (0.12)	5.98 (0.08)	< 0.001	2.70	6.36 (0.64)	52.08 (2.17)	< 0.001	2.09
25 months	3.59 (0.12)	6.28 (0.08)	< 0.001	2.86	5.23 (0.55)	53.54 (2.29)	< 0.001	2.10
26 months	3.74 (0.13)	6.59 (0.08)	< 0.001	2.96	4.26 (0.66)	55.15 (2.49)	< 0.001	2.03
27 months	3.89 (0.14)	6.89 (0.09)	< 0.001	2.81	3.45 (0.95)	56.93 (2.77)	< 0.001	1.91
28 months	4.05 (0.14)	7.20 (0.09)	< 0.001	2.95	2.86 (1.35)	58.88 (3.15)	< 0.001	1.75
29 months	4.20 (0.15)	7.50 (0.10)	< 0.001	2.81	2.43 (1.81)	60.99 (3.61)	< 0.001	1.59
30 months	4.35 (0.16)	7.81 (0.10)	< 0.001	2.89	2.16 (2.34)	63.26 (4.16)	< 0.001	1.43

*Note:* The estimated scores are for the “average child” (i.e., participants with average value on all covariates).

Abbreviations: *β* = unstandardized coefficient; SE = standard error.

## 4 Discussion

In this paper, we sought to explore whether and the extent to which productive vocabulary growth trajectories differed between LTs and their peers based on data from a low‐income sample of children between 8 and 30 months of age. Our analyses support that there are indeed distinctly different trajectories of vocabulary growth for LTs and their peers, such that: (1) LTs experience a relatively flat trajectory, resulting in a significantly smaller vocabulary size estimates by about age two, compared to the exponential growth experienced by their peers; and (2) the point of divergence occurs at ∼11 months of age.

These results support the previous finding that children in low‐income households are at heightened risk for language delay and disorder (e.g., Fisher 2017; Horwitz et al. 2003; Norbury et al. 2016), as 29% of our sample were identified as LTs compared to 10%–20% in socio‐demographically diverse epidemiological samples (American Speech‐Language‐Hearing Association 2024a; Zubrick et al. 2007). Notably, populations experiencing low income are historically underrepresented in longitudinal research, even in epidemiological and cohort studies focused on language delays (e.g., Collisson et al. 2016; Horwitz et al. 2003; Zubrick et al. 2007). Thus, part of this discrepancy may exist due to the lack of representation in previous studies. This paper is one of the first large‐scale longitudinal studies conducted in the U.S. to investigate language growth trajectories in an entirely low‐income sample and attempts to build an understanding of how low‐income LTs and non‐LTs exhibit differences in language trajectories during early childhood.

Our analyses also suggest that vocabulary size estimate growth significantly differs between LTs and their peers, such that LTs acquire vocabulary at a much slower rate than their peers and, as they age, the vocabulary gap between LTs and their peers increases exponentially. Given the striking differences in growth trajectories between LTs and their peers, it is no surprise that overall Words Produced sum scores differed greatly between the two groups at every time point, with LTs producing about ∼11% as many words as their peers. In our sample, at around 24 months, LTs had a mean Words Produced sum score of about 41 ± 33 words (median ≈ 34 words), while their peers had a Words Produced sum score of about 368 ± 187 words (median ≈ 342 words). When we compare this to published normative data (*N* > 6500 children), the median Words Produced sum score (i.e., 50^th^ percentile) for children at 24 months across a socioeconomically diverse group is 279 words (girls = 295 and boys = 226), while the 10^th^ percentile LT cut‐off for children is 66 words (girls = 74 and boys = 46; Marchman et al. 2023). These results further highlight the large discrepancies in vocabulary abilities between LTs and their peers in this sample, underscoring the need for early identification and intervention within this population.

Further research is needed to identify why vocabulary growth trajectories differ so dramatically between LTs and their peers, as this may point us in a direction for designing interventions suited to reducing these differences. While some characteristics that may be associated with LT risk cannot be intervened upon—for example: (1) child‐level socio‐demographics (e.g., sex, gestational age, birthweight; Collisson et al. 2016; Hammer et al. 2017; Hentges et al. 2019; Reilly et al. 2007; Zubrick et al. 2007); (2) parent‐level characteristics (e.g., family history of language delays, maternal education; Collisson et al. 2016; Hentges et al. 2019; Horwitz et al. 2003; Reilly et al. 2007; Zubrick et al. 2007); or (3) household‐level characteristics (e.g., household size, exposure to more than one language; Giesbrecht et al. 2024; Horwitz et al. 2003; Reilly et al. 2007; Zubrick et al. 2007)– these may represent areas that researchers and clinicians can hone in on to more promptly identify children who may be at risk of prolonged delay. Alternatively, exposure to less screen time (or at least higher quality screen time) (Giesbrecht et al. 2024; Madigan et al. 2020) and encouraging language comprehension and use of gesture (Verganti et al. 2024) are areas where interventions could be investigated for reducing LT risk, especially in populations experiencing low incomes.

Mother's psychological distress and/or parenting quality may better explain these differences in outcomes, following the theoretical approach of the Family Stress Model (Conger and Conger 2002; Masarik and Conger 2017) in understanding how economic pressure and hardship impact later child development outcomes (e.g., Singletary et al. 2025; Iruka et al. 2012; Perkins et al. 2013). Additionally, future research might focus on the types of words that LTs are failing to gain at the same rate as their non‐LT peers. As suggested by previous research, it may be that LTs are lacking specific word forms at certain times when their non‐LT peers are not, potentially based word learning biases (Jones 2003; Perry et al. 2022). For example, LTs may learn fewer shape‐based nouns (Perry et al. 2022), fewer manner verbs (Horvath et al. 2022), less phonological complex words (MacRoy‐Higgins et al. 2016) compared to their non‐LT age‐matched peers. Further, this could be related to the ways in which LTs differ from their age‐matched non‐LT peers in terms of how they use statistical patterns to expand their vocabulary, leading to a delayed trajectory (more like abilities‐matched younger children) rather than an objectively *different* learning trajectory (e.g., Simmons and Paul 2024). Understanding how LTs acquire words and how word‐learning biases may play a role into their language learning process should also be further investigated. For instance, during early word learning, Landau et al. (1988) found that both children and adults have a “shape bias” in which when presented with a novel word, they paired it with a new object based on its shape rather than its texture or size. However, shape bias and vocabulary development may occur differently in LTs compared to their peers. Weber and Colunga (2019) compared word learning biases and language trajectories of LTs and peers for 12 months. They found that at the initial visit at 16–18 months of age, for non‐LTs stronger shape bias had a positive relationship with a larger vocabulary size, yet a negative relationship was found for LTs. In subsequent visits, there was a positive relationship between shape bias and vocabulary for LTs. Further longitudinal research is needed to determine how word learning biases influence LTs language trajectories.

Previous research investigating language development in a sample of families experiencing low‐income with young children living in the same geographic area, the Kids in Columbus Study (KICS), points toward several associations that may help explain why LT and non‐LT children differ in their vocabulary growth trajectories during early childhood. For example, results from KICS suggest that variability in the early family context is associated with differences in child expressive and receptive language skill trajectories (using Bayley‐III) that emerge around age 24 months; specifically, having a mother with a lower degree of educational attainment was associated with lower language skill trajectories (Justice et al. 2020). Other variables related to educational attainment that may be worth looking into in future analyses—for example, economic hardship and/or parenting quality indicators. Another KICS study suggests that a combination of measures related to family stress, including economic hardship, parental well‐being, and parent‐child dysregulation interact to explain differences in receptive and expressive language skills in children in low‐income households (Justice et al. 2019). Additionally, exposure to environmental toxins within homes experiencing low income, regardless of sociodemographic variables, may also explain differences in language skills at age 2 (Jiang et al. 2020). Further, the quality of child‐directed talk in a parent‐child free play session, which tends to be correlated with maternal educational attainment, also predicts differences in receptive and expressive language skills in children in low‐income households at age 36 months old (Dore et al. 2022). Beyond the impacts of home context, attending childcare outside the home, particularly when you live in a high‐stress family, may be protective of language growth for receptive and expressive language skills (Dore et al. 2023). These results suggest directions for specific protective and risk factors that may help explain the differences in LT and non‐LT vocabulary growth trajectories within samples experiencing low income.

Our analyses suggest that ∼11 months is a critical period of divergence between LTs and non‐LTs in terms of language learning. Our results suggest that earlier identification and intervention may be necessary to reduce the risk of delayed vocabulary development (i.e., LT identification) and associated heightened risk of later developing DLD (e.g., Sansavini et al. 2021; Walters et al. 2020), versus being aided out of the LT category through strategic language intervention. However, we are not arguing that children should be getting screened by clinicians or researchers to be identified as LTs at this age. This age is in fact too early for LT identification, since the recommended age of assessment is when children are closer to 24 months and are expected to have larger vocabularies and should be combining words (e.g., Capone Singleton 2018; Collisson et al. 2016; Desmarais et al. 2008). Even identifying LT children at 18–23 months compared to 24–30 months is less stable (i.e., individuals are likely to shift from LT to non‐LT designation from one age to the next; Avelar et al. 2025), so trying to identify LTs at 11 months is not what this divergence point data suggests. It does, however, seem to align with earlier suggestions that LTs differ from the onset of expressive vocabulary forward (e.g., Ellis and Thal 2008) and provides more fine‐grained detail about their different learning trajectories. As Simmons and Paul (2024) suggest that LTs have delayed trajectories (matching their skills‐matched younger non‐LT peers), it is also possible that later LT growth trajectories (following identification at 24 months) begin to mimic those previously exhibited by their non‐LT peers, but on a delayed timescale—this would require future research with a larger timescale of longitudinal data. Notably, growth trajectories for LTs and their peers may behave differently in other SES groups; thus, the type of approach to understanding the shape and timing of growth trajectory divergence demonstrated in the current study must be repeated using longitudinal data collected from middle‐ and high‐SES families to better inform generalized policies about the identification and treatment of LT and DLD in young children across SES populations more generally.

Since our results suggest that there is a point of divergence between LTs and non‐LTs in terms of language learning ∼11 months, we highlight additional changes that can be captured at this age that may explain why this divergence occurred at this time, and how this could help researchers and clinicians implement earlier interventions better. Around this time, infants are becoming more mobile (e.g., walking while holding onto things), playing social games, and developing fine motor skills, all of which allows children to become more socially interactive and start to explore the world and people around them, generally encouraging the use of their first set of words around this age (Center for Disease Control 2023). Using looking‐while‐listening, eye‐tracking, and observational studies of differences in exploratory and motor behaviors may help us better understand how LT and non‐LT peers begin to differ around their first year. For example, even at age 6–9 months, prior to producing any expressed language, a study of 33 infants reveals that differences in language comprehension of common nouns (e.g., body parts and foods) can be observed using looking‐while‐listening tasks, and that infants as young as 6 months old may be comprehending the connection between words and their associated objects as shown in pictures (Bergelson and Swingley 2012). As some degree of language comprehension is generally necessary prior to the production of first words (Carpenter et al. 1998), understanding whether there are differences in early comprehension in LT versus non‐LT infants would be an interesting avenue of new research. Further, in a study of 28 LT and non‐LTs, an application of the looking‐while‐listening protocol (using eye‐tracking methods) revealed that LT children being to show differences in their moment‐by‐moment processing of novel words at around 18 months, such that LTs exhibit patterns that may coincide with difficulty to learning new words as compared to their non‐LT peers (Ellis et al. 2015). An additional eye‐tracking study of 22 two‐year‐olds suggest that LT toddlers allocate less attention during novel word learning, and that these differences may contribute to their overall productive vocabulary growth delay (MacRoy‐Higgins and Montemarano 2016). Thus, early implementation during infancy of eye‐tracking studies may reveal evidence of similar processing differences that could be intervened upon to reduce negative outcomes (e.g., helping tune the attention of potential LT children during word learning).

Further research is needed to fully understand LT growth trajectories in terms of how they differ from their non‐LT peers, why this divergence point may emerge at ∼11 months, and how this timing is related to underlaying language learning processes. However, as far as we know, this was the first large‐scale longitudinal study to examine this data in early infancy. Notably, it is possible that the results we present here are only part of the earliest picture of these growth trajectories, and that if we had similar vocabulary data at later ages to continue following these children at later TPs, we might be able to zoom out and see that rather than exhibiting starkly different trajectories, LTs follow a delayed but similarly shaped trajectory to their non‐LT peers (e.g., Simmons and Paul 2024), and that the biggest difference in LT and non‐LT trajectories is the point in time at which exponential growth occurs. Nonetheless, this is one of the first studies to compare LTs and their non‐LT peers exclusively in a sample of U.S. families experiencing low income. This research thus fills a gap in the current literature and suggests important areas for future research. As children growing up in homes experiencing low income are at particularly heightened risk for LT and later DLD, this research marks an important step forward toward helping improve our ability to approach these SES‐based discrepancies in language development.

### 4.1 Limitations

The present study has several limitations. First, the results presented here cannot be generalized to a larger population, as they are drawn from a convenience sample. Second, there were missing data and attrition across timepoints which complicates our ability to analyze growth over time for LTs and their peers. We attribute these issues to the longitudinal nature of SMALL Talk and the involvement of a historically marginalized population, who face unique challenges to continued participation in such research. However, despite these issues that impact the overall sample size of our analyses, this data is of high importance to discuss, as historically marginalized populations also tend to have some of the highest LT rates. Third, as child productive vocabulary scores analyzed here are derived from self‐reported questionnaires filled out by mothers (vs. observer‐rated tests of child vocabulary production in the field), this may impact the overall reliability of our findings. The use of assessor‐rated measures of child language skills collected at later timepoints from SMALL Talk participants may provide a more nuanced picture of the ways in which vocabulary growth trajectories differ between LTs and their peers in a sample of children in low‐income households.

Finally, in our model, we use LT status as determined by CDI assessed at TP4 as an outcome, while using vocabulary size estimates derived from CDI at TP1‐4 to model developmental trajectories—these scores are arguably highly related. However, because we were interested in answering the question of whether and to what extent the developmental trajectories differ in the vocabulary size estimates from TP1 to TP4, for those scored above a threshold for LT in TP4 (i.e., non‐LTs) versus those below the threshold in TP4 (i.e., LTs), there is no statistical artifact effect on the results of our model. Our model does not automatically assume anything about the *changes* of vocabulary size estimates from TP1 to TP4. However, these results should be interpreted with some degree of caution, as we did use CDI in identification of LT status, as well as to generate vocabulary size estimates for trajectory evaluation. Future research should attempt to reproduce these results using a separate measure of LT status (e.g., an assessor‐rated measure of language skills using a clinical measure such as the Preschool Language Scales [Zimmerman et al. 2011] and/or Clinical Evaluation of Language Fundamentals [Wiig et al. 2013]) to map vocabulary growth trajectory at earlier timepoints. Our dataset is limited by the lack of a single vocabulary assessment carried out across all TPs of the study, which makes this type of longitudinal analysis more complex. However, expanding the model to include language measures from TPs when children are older may enable us to more accurately determine whether LT and non‐LT growth trajectories are truly *different* or if LTs experience markedly *delayed* growth trajectories (but with a similar shape) in comparison to their peers (e.g., Simmons and Paul 2024).

## 5 Conclusion

The current study had two aims: (1) to examine vocabulary growth over time to determine whether LTs show a *delayed* growth trajectory or a *different* growth trajectory, compared to non‐LT peers; and (2) to identify the age at which LT and non‐LT growth trajectories diverge. We used multi‐level multiple group models to map out the vocabulary size estimate growth trajectories for LTs and their non‐LT peers and to pinpoint the age at which a clear divergence in vocabulary size estimate occurred. We found that vocabulary growth trajectories of LTs were relatively flat compared to the exponential growth experienced by non‐LT peers, and that these differently‐shaped trajectories diverged at ∼11 months of age. If LTs and their peers generally begin to significantly diverge in vocabulary size at ∼11 months of age, it is important that we determine exactly how they differ from their non‐LT peers (in terms of types of words, word classes, or neighborhood densities, e.g., Simmons and Paul 2024), why this divergence point may emerge at ∼11 months, and how this timing is related to underlaying language learning processes. Furthermore, this work should be extended to larger populations, including the full array of SES‐statuses to more generally understand these processes leading to differences in vocabulary growth within LT children.

## Author Contributions

**Britt Singletary:** conceptualization, data curation, investigation, methodology, project administration, supervision, writing – original draft, writing – review and editing. **Hui Jiang:** formal analysis, supervision, writing – original draft, writing – review and editing. **Winifred Graham Wilberforce:** data curation, formal analysis, writing – original draft, writing – review and editing. **Daniela Avelar:** data curation, writing – original draft, writing – review and editing. **Kristina Strother‐Garcia:** writing – original draft, writing – review and editing. **Laura M. Justice:** conceptualization, funding acquisition, supervision, writing – review and editing.

## Conflicts of Interest

The authors declare no conflicts of interest.

## Supporting information

Table S1

Table S2

## Data Availability

The datasets generated during and/or analyzed during the current study are available from the corresponding author on reasonable request.

## References

[infa70036-bib-0001] American Speech‐Language‐Hearing Association . 2024a. Late Language Emergence, March 4. https://www.asha.org/practice‐portal/clinical‐topics/late‐language‐emergence/.

[infa70036-bib-0002] American Speech‐Language‐Hearing Association . 2024b. Spoken Language Disorders, March 4. https://www.asha.org/practice‐portal/clinical‐topics/spoken‐language‐disorders/.

[infa70036-bib-0003] Anderson, N. J. , S. A. Graham , H. Prime , J. M. Jenkins , and S. Madigan . 2021. “Linking Quality and Quantity of Parental Linguistic Input to Child Language Skills: A Meta‐Analysis.” Child Development 92, no. 2: 484–501. 10.1111/cdev.13508. 33521953

[infa70036-bib-0004] Armstrong, R. , J. G. Scott , A. J. Whitehouse , D. A. Copland , K. L. McMahon , and W. Arnott . 2017. “Late Talkers and Later Language Outcomes: Predicting the Different Language Trajectories.” International Journal of Speech Language Pathology 19, no. 3: 237–250. 10.1080/17549507.2017.1296191. 28440674

[infa70036-bib-0005] Arriaga, R. I. , L. Fenson , T. Cronan , and S. J. Pethick . 1998. “Scores on the MacArthur Communicative Development Inventory of Children From Low‐ and Middle‐Income Families.” Applied PsychoLinguistics 19, no. 2: 209–223. 10.1017/S0142716400010043.

[infa70036-bib-0120] Avelar, D. , B. Singletary , P. S. Dale , and L. M. Justice . 2025. “The Impact of Diverse Parameters for Late Talker Identification in a Low–Socioeconomic Status Sample.” Journal of Speech, Language, and Hearing Research 68, no. 05: 2453–2467. 10.1044/2025_jslhr-24-00637. 40268732

[infa70036-bib-0010] Beckage, N. , L. Smith , T. Hills , and M. Perc . 2011. “Small Worlds and Semantic Network Growth in Typical and Late Talkers.” PLoS One 6, no. 5: e19348. 10.1371/journal.pone.0019348. 21589924 PMC3092758

[infa70036-bib-0011] Bergelson, E. , and D. Swingley . 2012. “At 6–9 Months, Human Infants Know the Meanings of Many Common Nouns.” Proceedings of the National Academy of Sciences 109, no. 9: 3253–3258. 10.1073/pnas.1113380109. PMC3295309 22331874

[infa70036-bib-0012] Berger, L. M. , C. Paxson , and J. Waldfogel . 2009. “Income and Child Development.” Children and Youth Services Review 31, no. 9: 978–989. 10.1016/j.childyouth.2009.04.013. 20368763 PMC2847736

[infa70036-bib-0013] Bishop, D. V. M. , M. J. Snowling , P. A. Thompson , and T. Greenhalgh . 2017. “Phase 2 of CATALISE: A Multinational and Multidisciplinary Delphi Consensus Study of Problems With Language Development: Terminology.” Journal of Child Psychology and Psychiatry 58, no. 10: 1068–1080. 10.1111/jcpp.12721. 28369935 PMC5638113

[infa70036-bib-0014] Bleses, D. , and W. Vach . 2013. “Danish Late Talkers: A First Portrait.” In Late Talkers: From Research to Practice, edited by L. A. Rescorla and P. S. Dale , 41–65. Brookes Publishing.

[infa70036-bib-0015] Buac, M. , M. Gross , and M. Kaushanskaya . 2014. “The Role of Primary Caregiver Vocabulary Knowledge in the Development of Bilingual Children's Vocabulary Skills.” Journal of Speech, Language, and Hearing Research 57, no. 5: 1804–1816. 10.1044/2014_JSLHR-L-13-0055. PMC4632841 24824882

[infa70036-bib-0016] Capone Singleton, N. 2018. “Late Talkers: Why the Wait‐and‐See Approach Is Outdated.” Pediatric Clinics of North America 65, no. 1: 13–29. 10.1016/j.pcl.2017.08.018. 29173713

[infa70036-bib-0017] Carpenter, M. , K. Nagell , M. Tomasello , G. Butterworth , and C. Moore . 1998. “Social Cognition, Joint Attention, and Communicative Competence From 9 to 15 Months of Age.” Monographs of the Society for Research in Child Development 63, no. 4: i‐174. 10.2307/1166214. 9835078

[infa70036-bib-0018] Carson, C. P. , T. Klee , D. K. Carson , and L. K. Hime . 2003. “Phonological Profiles of 2‐Year‐Olds With Delayed Language Development: Predicting Clinical Outcomes at Age 3.” American Journal of Speech‐Language Pathology 12, no. 1: 28–39. 10.1044/1058-0360(2003/050). 12680811

[infa70036-bib-0019] Center for Disease Control . 2023. Important Milestones: Your Child by One Year. https://www.cdc.gov/ncbddd/actearly/milestones/milestones‐1yr.html.

[infa70036-bib-0020] Cheung, R. W. , K. Willan , J. Dickerson , and C. Bowyer‐Crane . 2023. “Risk Factors for Early Language Delay in Children Within a Minority Ethnic, Bilingual, Deprived Environment (Born in Bradford’s Better Start): A UK Community Birth Cohort Study.” BMJ Paediatrics Open 7, no. 1: e001764. 10.1136/bmjpo-2022-001764. 36927864 PMC10030670

[infa70036-bib-0021] Collins, L. M. , J. L. Schafer , and C. M. Kam . 2001. “A Comparison of Inclusive and Restrictive Strategies in Modern Missing Data Procedures.” Psychological Methods 6, no. 4: 330–351. 10.1037/1082-989x.6.4.330. 11778676

[infa70036-bib-0022] Collisson, B. A. , S. A. Graham , J. L. Preston , M. S. Rose , S. McDonald , and S. Tough . 2016. “Risk and Protective Factors for Late Talking: An Epidemiologic Investigation.” Journal of Pediatrics 172: 168–174.e161. 10.1016/j.jpeds.2016.02.020. 26968834

[infa70036-bib-0023] Conger, R. D. , and K. J. Conger . 2002. “Resilience in Midwestern Families: Selected Findings From the First Decade of a Prospective, Longitudinal Study.” Journal of Marriage and Family 64, no. 2: 361–373. 10.1111/j.1741-3737.2002.00361.x.

[infa70036-bib-0024] Cook, M. L. , J. J. Yan , and K. Butler . 2024. “Maternal Parenting Stress and Child Externalizing Behaviors: Low‐Income as a Context.” Journal of Applied Developmental Psychology 93: 101673. 10.1016/j.appdev.2024.101673.

[infa70036-bib-0025] Dale, P. S. , T. S. Price , D. V. Bishop , and R. Plomin . 2003. “Outcomes of Early Language Delay: Part I. Predicting Persistent and Transient Language Difficulties at 3 and 4 Years.” Journal of Speech, Language, and Hearing Research 46, no. 3: 544–560. 10.1044/1092-4388(2003/044). 14696985

[infa70036-bib-0026] Desmarais, C. , A. Sylvestre , F. Meyer , I. Bairati , and N. Rouleau . 2008. “Systematic Review of the Literature on Characteristics of Late‐Talking Toddlers.” International Journal of Language & Communication Disorders 43, no. 4: 361–389. 10.1080/13682820701546854. 17885825

[infa70036-bib-0027] Dore, R. A. , X. Liu , L. J. Chaparro‐Moreno , and L. M. Justice . 2022. “Concurrent Relations Between Child‐Directed Speech and Children’s Language Skills in Low‐Income Households.” Journal of Early Childhood Research 20, no. 4: 479–494. 10.1177/1476718X221098661.

[infa70036-bib-0028] Dore, R. A. , K. M. Purtell , J. Chen , and L. M. Justice . 2023. “The Interplay Among Parents’ Stress, Nonparental Childcare, and Child Language Development Among Low‐Income Toddlers.” Early Education & Development 34, no. 6: 1447–1457. 10.1080/10409289.2022.2106767.

[infa70036-bib-0029] Dubois, P. , M.‐C. St‐Pierre , C. Desmarais , and F. Guay . 2020. “Young Adults With Developmental Language Disorder: A Systematic Review of Education, Employment, and Independent Living Outcomes.” Journal of Speech, Language, and Hearing Research 63, no. 11: 3786–3800. 10.1044/2020_JSLHR-20-00127. 33022192

[infa70036-bib-0030] Ellis, E. M. , A. Borovsky , J. L. Elman , and J. L. Evans . 2015. “Novel Word Learning: An Eye‐Tracking Study. Are 18‐Month‐Old Late Talkers Really Different From Their Typical Peers?” Journal of Communication Disorders 58: 143–157. 10.1016/j.jcomdis.2015.06.011. 26188415 PMC4659719

[infa70036-bib-0031] Ellis, E. M. , and D. J. Thal . 2008. “Early Language Delay and Risk for Language Impairment.” Perspectives on Language Learning and Education 15, no. 3: 93–100. 10.1044/lle15.3.93.

[infa70036-bib-0032] Fenson, L. , P. S. Dale , J. S. Reznick , et al. 1993. MacArthur Communicative Development Inventories: User’s Guide and Technical Manual. Singular Publishing Group.

[infa70036-bib-0033] Fenson, L. , P. S. Dale , J. S. Reznick , et al. 1994. “Variability in Early Communicative Development.” Monographs of the Society for Research in Child Development 59, no. 5: i‐185. 10.2307/1166093. 7845413

[infa70036-bib-0034] Fenson, L. , V. A. Marchman , D. Thal , P. Dale , J. S. Reznick , and E. Bates . 2007. MacArthur Communicative Development Inventories: User's Guide and Technical Manual. 2nd ed. Paul H. Brookes Publishing Company.

[infa70036-bib-0035] Fisher, E. L. 2017. “A Systematic Review and Meta‐Analysis of Predictors of Expressive‐Language Outcomes Among Late Talkers.” Journal of Speech, Language, and Hearing Research 60, no. 10: 2935–2948. 10.1044/2017_JSLHR-L-16-0310. 28915512

[infa70036-bib-0036] Giesbrecht, G. F. , M. van de Wouw , D. Watts , et al. 2024. “Language Learning in the Context of a Global Pandemic: Proximal and Distal Factors Matter.” Pediatric Research 97, no. 6: 1–11. 10.1038/s41390-024-03583-9. 39294240

[infa70036-bib-0037] Golinkoff, R. M. , E. Hoff , M. L. Rowe , C. S. Tamis‐LeMonda , and K. Hirsh‐Pasek . 2019. “Language Matters: Denying the Existence of the 30‐Million‐Word Gap Has Serious Consequences.” Child Development 90, no. 3: 985–992. 10.1111/cdev.13128. 30102419 PMC10370358

[infa70036-bib-0038] Hammer, C. S. , P. Morgan , G. Farkas , M. Hillemeier , D. Bitetti , and S. Maczuga . 2017. “Late Talkers: A Population‐Based Study of Risk Factors and School Readiness Consequences.” Journal of Speech, Language, and Hearing Research 60, no. 3: 607626. 10.1044/2016_JSLHR-L-15-0417. PMC5962923 28257586

[infa70036-bib-0039] Hart, B. , and R. R. Risely . 1995. Meaningful Differences in the Everyday Experiences of Young American Children. Paul H. Brooks Publishing.

[infa70036-bib-0040] Hawa, V. V. , and G. Spanoudis . 2014. “Toddlers With Delayed Expressive Language: An Overview of the Characteristics, Risk Factors and Language Outcomes.” Research in Developmental Disabilities 35, no. 2: 400–407. 10.1016/j.ridd.2013.10.027. 24334229

[infa70036-bib-0041] Henrichs, J. , L. Rescorla , J. J. Schenk , et al. 2011. “Examining Continuity of Early Expressive Vocabulary Development: The Generation R Study.” Journal of Speech, Language, and Hearing Research 54, no. 3: 854–869. 10.1044/1092-4388(2010/09-0255). 20966386

[infa70036-bib-0042] Hentges, R. F. , S. Madigan , A. Plamondon , et al. 2019. “Heterogeneous Trajectories of Delayed Communicative Development From 12 to 36 Months: Predictors and Consequences.” Journal of Developmental and Behavioral Pediatrics 40, no. 5: 335–343. 10.1097/DBP.0000000000000677. 31206452

[infa70036-bib-0043] Hirsh‐Pasek, K. , L. B. Adamson , R. Bakeman , et al. 2015. “The Contribution of Early Communication Quality to Low‐Income Children’s Language Success.” Psychological Science 26, no. 7: 1071–1083. 10.1177/0956797615581493. 26048887

[infa70036-bib-0044] Hoff, E. 2003. “The Specificity of Environmental Influence: Socioeconomic Status Affects Early Vocabulary Development via Maternal Speech.” Child Development 74, no. 5: 1368–1378. 10.1111/1467-8624.00612. 14552403

[infa70036-bib-0045] Hoff, E. 2013. “Interpreting the Early Language Trajectories of Children From Low‐SES and Language Minority Homes: Implications for Closing Achievement Gaps.” Developmental Psychology 49, no. 1: 4–14. 10.1037/a0027238. 22329382 PMC4061698

[infa70036-bib-0046] Hoff, E. , and L. Naigles . 2002. “How Children Use Input to Acquire a Lexicon.” Child Development 73, no. 2: 418–433. 10.1111/1467-8624.00415. 11949900

[infa70036-bib-0047] Hoff‐Ginsberg, E. 1998. “The Relation of Birth Order and Socioeconomic Status to Children’s Language Experience and Language Development.” Applied PsychoLinguistics 19, no. 4: 603–629. 10.1017/S0142716400010389.

[infa70036-bib-0048] Horvath, S. , J. B. Kueser , J. Kelly , and A. Borovsky . 2022. “Difference or Delay? Syntax, Semantics, and Verb Vocabulary Development in Typically Developing and Late‐Talking Toddlers.” Language Learning and Development 18, no. 3: 352–376. 10.1080/15475441.2021.1977645. 35664680 PMC9159542

[infa70036-bib-0049] Horwitz, S. M. , J. R. Irwin , M. J. Briggs‐Gowan , J. M. Bosson Heenan , J. Medoza , and A. S. Carter . 2003. “Language Delay in a Community Cohort of Young Children.” Journal of the American Academy of Child & Adolescent Psychiatry 42, no. 8: 932–940. 10.1097/01.CHI.0000046889.27264.5E. 12874495

[infa70036-bib-0050] Hsu, N. , P. A. Hadley , and M. Rispoli . 2017. “Diversity Matters: Parent Input Predicts Toddler Verb Production.” Journal of Child Language 44, no. 1: 63–86. 10.1017/S0305000915000690. 26638832

[infa70036-bib-0051] Huttenlocher, J. , W. Haight , A. Bryk , M. Seltzer , and T. Lyons . 1991. “Early Vocabulary Growth: Relation to Language Input and Gender.” Developmental Psychology 27, no. 2: 236–248. 10.1037/0012-1649.27.2.236.

[infa70036-bib-0052] Iruka, I. U. , D. R. LaForett , and E. C. Odom . 2012. “Examining the Validity of the Family Investment and Stress Models and Relationship to Children's School Readiness Across Five Cultural Groups.” Journal of Family Psychology 26, no. 3: 359–370. 10.1037/a0028290. 22545934

[infa70036-bib-0053] Jiang, H. , L. M. Justice , K. M. Purtell , and R. Bates . 2020. “Exposure to Environmental Toxicants and Early Language Development for Children Reared in Low‐Income Households.” Clinical Pediatrics 59, no. 6: 557–565. 10.1177/0009922820908591. 32107933 PMC9811599

[infa70036-bib-0054] Jones, S. S. 2003. “Late Talkers Show No Shape Bias in a Novel Name Extension Task.” Developmental Science 6, no. 5: 477–483. 10.1111/1467-7687.00304.

[infa70036-bib-0055] Justice, L. M. , H. Jiang , R. Bates , and A. Koury . 2020. “Language Disparities Related to Maternal Education Emerge by Two Years in a Low‐Income Sample.” Maternal and Child Health Journal 24, no. 11: 1419–1427. 10.1007/s10995-020-02973-9. 32632843 PMC7572544

[infa70036-bib-0056] Justice, L. M. , H. Jiang , K. M. Purtell , et al. 2019. “Conditions of Poverty, Parent–Child Interactions, and Toddlers’ Early Language Skills in Low‐Income Families.” Maternal and Child Health Journal 23, no. 7: 971–978. 10.1007/s10995-018-02726-9. 30649661 PMC6778955

[infa70036-bib-0119] Justice, L. M. , B. Singletary , H. Jiang , and K. K. Schmeer . 2025. “Profiles of Family Stressors Among Low‐Income Families With Young Children.” Maternal and Child Health Journal 29, no. 4: 483–493. 10.1007/s10995-025-04061-2. 40000565 PMC12006244

[infa70036-bib-0057] Korpilahti, P. , A. Kaljonen , and E. Jansson‐Verkasalo . 2016. “Population‐Based Screening for Language Delay: Let’s Talk STEPS Study.” Psychology 7, no. 2: 205–214. 10.4236/psych.2016.72023. 26700576

[infa70036-bib-0058] Landau, B. , L. B. Smith , and S. S. Jones . 1988. “The Importance of Shape in Early Lexical Learning.” Cognitive Development 3, no. 3: 299–321. 10.1016/0885-2014(88)90014-7.

[infa70036-bib-0059] MacRoy‐Higgins, M. , and E. A. Montemarano . 2016. “Attention and Word Learning in Toddlers Who Are Late Talkers.” Journal of Child Language 43, no. 5: 1020–1037. 10.1017/S0305000915000379. 27464621

[infa70036-bib-0060] MacRoy‐Higgins, M. , V. L. Shafer , K. J. Fahey , and E. R. Kaden . 2016. “Vocabulary of Toddlers Who Are Late Talkers.” Journal of Early Intervention 38, no. 2: 118–129. 10.1177/1053815116637620.

[infa70036-bib-0061] Madigan, S. , B. A. McArthur , C. Anhorn , R. Eirich , and D. A. Christakis . 2020. “Associations Between Screen Use and Child Language Skills: A Systematic Review and Meta‐Analysis.” JAMA Pediatrics 174, no. 7: 665–675. 10.1001/jamapediatrics.2020.0327. 32202633 PMC7091394

[infa70036-bib-0062] Madigan, S. , H. Prime , S. A. Graham , et al. 2019. “Parenting Behavior and Child Language: A Meta‐Analysis.” Pediatrics 144, no. 4: e20183556. 10.1542/peds.2018-3556. 31551396

[infa70036-bib-0063] Manhardt, J. , and L. Rescorla . 2002. “Oral Narrative Skills of Late Talkers at Ages 8 and 9.” Applied PsychoLinguistics 23, no. 1: 1–21. 10.1017/s0142716402000012.

[infa70036-bib-0064] Marchman, V. , P. Dale , and L. Fenson . 2023. MacArthur‐Bates Communicative Development Inventories: User’s Guide and Technical Manual. 3rd ed. Brookes Publishing.

[infa70036-bib-0065] Masarik, A. S. , and R. D. Conger . 2017. “Stress and Child Development: A Review of the Family Stress Model.” Current Opinion in Psychology 13: 85–90. 10.1016/j.copsyc.2016.05.008. 28813301

[infa70036-bib-0066] Mayor, J. , and K. Plunkett . 2010. Vocabulary Size Calculator – Basque Center on Cognition, Brain, and Language (Applet). https://www.bcbl.eu/cdi/.

[infa70036-bib-0067] Mayor, J. , and K. Plunkett . 2011. “A Statistical Estimate of Infant and Toddler Vocabulary Size From CDI Analysis.” Developmental Science 14, no. 4: 769–785. 10.1111/j.1467-7687.2010.01024.x. 21676097

[infa70036-bib-0068] Miller, P. J. , D. E. Sperry , and L. L. Sperry . 2024. “A Deficit Story in Motion: How Marginalized Youngsters Are Defined Out of the Educational Game Before They Enter School.” Journal of Social Issues 80, no. 4: 1218–1237. 10.1111/josi.12647.

[infa70036-bib-0069] Morgan, L. , A. Delehanty , J. Cleary Dillon , C. Schatschneider , and A. M. Wetherby . 2020. “Measures of Early Social Communication and Vocabulary Production to Predict Language Outcomes at Two and Three Years in Late‐Talking Toddlers.” Early Childhood Research Quarterly 51: 366–378. 10.1016/j.ecresq.2019.12.005. 32863566 PMC7455001

[infa70036-bib-0070] Muthén, L. K. , and B. O. Muthén . 1998–2017. Mplus User’s Guide. 8th ed. Muthén & Muthén.

[infa70036-bib-0071] Norbury, C. F. , D. Gooch , C. Wray , et al. 2016. “The Impact of Nonverbal Ability on Prevalence and Clinical Presentation of Language Disorder: Evidence From a Population Study.” Journal of Child Psychology and Psychiatry 57, no. 11: 1247–1257. 10.1111/jcpp.12573. 27184709 PMC5082564

[infa70036-bib-0072] Pace, A. , R. Luo , K. Hirsh‐Pasek , and R. M. Golinkoff . 2017. “Identifying Pathways Between Socioeconomic Status and Language Development.” Annual Review of Linguistics 3, no. 2017: 285–308. 10.1146/annurev-linguistics-011516-034226.

[infa70036-bib-0073] Paul, R. 1993. “Patterns of Development in Late Talkers: Preschool Years.” Journal of Childhood Communication Disorders 15, no. 1: 7–14. 10.1177/152574019301500103.

[infa70036-bib-0074] Paul, R. , and S. E. Weismer . 2013. “Late Talking in Context: The Clinical Implications of Delayed Language Development.” In Late Talkers: Language Development, Interventions, and Outcomes, edited by L. Rescorla and P. S. Dale , 203–217. Brookes Publishing.

[infa70036-bib-0075] Perkins, S. C. , E. D. Finegood , and J. E. Swain . 2013. “Poverty and Language Development: Roles of Parenting and Stress.” Innovations in Clinical Neuroscience 10, no. 4: 10–19. PMC3659033 23696954

[infa70036-bib-0076] Perry, L. K. , S. C. Kucker , J. S. Horst , and L. K. Samuelson . 2022. “Late Bloomer or Language Disorder? Differences in Toddler Vocabulary Composition Associated With Long‐Term Language Outcomes.” Developmental Science 26, no. 4: e13342. 10.1111/desc.13342. 36354235

[infa70036-bib-0077] Poll, G. H. , and C. A. Miller . 2013. “Late Talking, Typical Talking, and Weak Language Skills at Middle Childhood.” Learning and Individual Differences 26: 177–184. 10.1016/j.lindif.2013.01.008. 24039376 PMC3768009

[infa70036-bib-0078] Preston, J. L. , S. J. Frost , W. E. Mencl , et al. 2010. “Early and Late Talkers: School‐Age Language, Literacy and Neurolinguistic Differences.” Brain 133, no. 8: 2185–2195. 10.1093/brain/awq163. 20826428 PMC3139938

[infa70036-bib-0079] Pungello, E. P. , I. U. Iruka , A. M. Dotterer , R. Mills‐Koonce , and J. S. Reznick . 2009. “The Effects of Socioeconomic Status, Race, and Parenting on Language Development in Early Childhood.” Developmental Psychology 45, no. 2: 544–557. 10.1037/a0013917. 19271838

[infa70036-bib-0080] Purpura, D. J. 2019. “Language Clearly Matters: Methods Matter Too.” Child Development 90, no. 6: 1839–1846. 10.1111/cdev.13327. 31625601

[infa70036-bib-0081] Quiroz, B. G. , C. E. Snow , and J. Zhao . 2010. “Vocabulary Skills of Spanish‐English Bilinguals: Impact of Mother‐Child Language Interactions and Home Language and Literacy Support.” International Journal of Bilingualism 14, no. 4: 379–399. 10.1177/1367006910370919.

[infa70036-bib-0082] Raz, M. , and B. R. Beatty . 2018. “Replacing the ‘Word Gap’ With Nonstigmatizing Approaches to Early Literacy and Language Building.” Pediatrics 142, no. 6: e20181992. 10.1542/peds.2018-1992. 30389714

[infa70036-bib-0083] Reilly, S. , M. Wake , E. L. Bavin , et al. 2007. “Predicting Language at 2 Years of Age: A Prospective Community Study.” Pediatrics 120, no. 6: e1441–e1449. 10.1542/peds.2007-0045. 18055662

[infa70036-bib-0084] Reilly, S. , M. Wake , O. C. Ukoumunne , et al. 2011. “Predicting Language Outcomes at 4 Years of Age: Findings From Early Language in Victoria Study.” Pediatrics 126, no. 6: e1530–e1537. 10.1542/peds.2010-0254. 21059719

[infa70036-bib-0085] Rescorla, L. 1989. “The Language Development Survey: A Screening Tool for Delayed Language in Toddlers.” Journal of Speech and Hearing Disorders 54, no. 4: 587–599. 10.1044/jshd.5404.587. 2811339

[infa70036-bib-0086] Rescorla, L. 2005. “Age 13 Language and Reading Outcomes in Late‐Talking Toddlers.” Journal of Speech, Language, and Hearing Research 48, no. 2: 459–472. 10.1044/1092-4388(2005/031). 15989404

[infa70036-bib-0087] Rescorla, L. 2009. “Age 17 Language and Reading Outcomes in Late‐Talking Toddlers: Support for a Dimensional Perspective on Language Delay.” Journal of Speech, Language, and Hearing Research 52, no. 1: 16–30. 10.1044/1092-4388(2008/07-0171). 18723598

[infa70036-bib-0088] Rescorla, L. 2011. “Late Talkers: Do Good Predictors of Outcome Exist?” Developmental Disabilities Research Reviews 17, no. 2: 141–150. 10.1002/ddrr.1108. 23362033

[infa70036-bib-0089] Rescorla, L. , and A. Alley . 2001. “Validation of the Language Development Survey (LDS): A Parent Report Tool for Identifying Language Delay in Toddlers.” Journal of Speech, Language, and Hearing Research 44, no. 2: 434–445. 10.1044/1092-4388(2001/035). 11324663

[infa70036-bib-0090] Rescorla, L. , M. Hadicke‐Wiley , and E. Escarce . 1993. “Epidemiological Investigation of Expressive Language Delay at Age Two.” First Language 13, no. 37: 5–22. 10.1177/014272379301303702.

[infa70036-bib-0091] Rescorla, L. , J. Mirak , and L. Singh . 2000. “Vocabulary Growth in Late Talkers: Lexical Development From 2;0 to 3;0.” Journal of Child Language 27, no. 2: 293–311. 10.1017/s030500090000413x. 10967889

[infa70036-bib-0092] Rice, M. L. 2020. “Clinical Lessons From Studies of Children With Specific Language Impairment.” Perspectives of the ASHA Special Interest Groups 5, no. 1: 12–29. 10.1044/2019_PERSP-19-00011.

[infa70036-bib-0093] Rice, M. L. , C. L. Taylor , and S. R. Zubrick . 2008. “Language Outcomes of 7‐Year‐Old Children With or Without a History of Late Language Emergence at 24 Months.” Journal of Speech, Language, and Hearing Research 51, no. 2: 394–407. 10.1044/1092-4388(2008/029). 18367685

[infa70036-bib-0094] Rice, M. L. , S. R. Zubrick , C. L. Taylor , J. Gayán , and D. E. Bontempo . 2014. “Late Language Emergence in 24‐Month‐Old Twins: Heritable and Increased Risk for Late Language Emergence in Twins.” Journal of Speech, Language, and Hearing Research 57, no. 3: 917–928. 10.1044/1092-4388(2013/12-0350). PMC3975649 24167238

[infa70036-bib-0095] Roulstone, S. , S. Loader , K. Northstone , M. Beveridge , and the ALSPAC Team . 2002. “The Speech and Language of Children Aged 25 Months: Descriptive Data From the Avon Longitudinal Study of Parents and Children.” Early Child Development and Care 172, no. 3: 259–268. 10.1080/03004430212126.

[infa70036-bib-0096] Rowe, M. L. 2008. “Child‐Directed Speech: Relation to Socioeconomic Status, Knowledge of Child Development and Child Vocabulary Skill.” Journal of Child Language 35, no. 1: 185–205. 10.1017/S0305000907008343. 18300434

[infa70036-bib-0097] Rowe, M. L. 2012. “A Longitudinal Investigation of the Role of Quantity and Quality of Child‐Directed Speech in Vocabulary Development.” Child Development 83, no. 5: 1762–1774. 10.1111/j.1467-8624.2012.01805.x. 22716950 PMC3440540

[infa70036-bib-0098] Rowe, M. L. , and C. E. Snow . 2020. “Analyzing Input Quality Along Three Dimensions: Interactive, Linguistic, and Conceptual.” Journal of Child Language 47, no. 1: 5–21. 10.1017/S0305000919000655. 31668157

[infa70036-bib-0099] Sansavini, A. , M. E. Favilla , M. T. Guasti , et al. 2021. “Developmental Language Disorder: Early Predictors, Age for the Diagnosis, and Diagnostic Tools. A Scoping Review.” Brain Sciences 11, no. 5: 654. 10.3390/brainsci11050654. 34067874 PMC8156743

[infa70036-bib-0100] Satorra, A. , and P. M. Bentler . 2010. “Ensuring Positiveness of the Scaled Difference Chi‐Square Test Statistic.” Psychometrika 75, no. 2: 243–248. 10.1007/s11336-009-9135-y. 20640194 PMC2905175

[infa70036-bib-0101] Schafer, J. L. , and J. W. Graham . 2002. “Missing Data: Our View of the State of the Art.” Psychological Methods 7, no. 2: 147–177. 10.1037/1082-989X.7.2.147. 12090408

[infa70036-bib-0102] Schjølberg, S. , P. Eadie , H. D. Zachrisson , A.‐S. Øyen , and M. Prior . 2011. “Predicting Language Development at Age 18 Months: Data From the Norwegian Mother and Child Cohort Study.” Journal of Developmental and Behavioral Pediatrics 32, no. 5: 375–383. 10.1097/DBP.0b013e31821bd1dd. 21546853

[infa70036-bib-0103] Schwab, J. F. , and C. Lew‐Williams . 2016. “Language Learning, Socioeconomic Status, and Child‐Directed Speech.” WIREs: Cognitive Science 7, no. 4: 264–275. 10.1002/wcs.1393. 27196418 PMC5901657

[infa70036-bib-0104] Sege, R. , D. Burstein , and C. Yang . 2022. “Responding to the Community: HOPE (Healthy Outcomes From Positive Experiences).” In Broadly Engaged Team Science in Clinical and Translational Research, edited by D. Lerner , M. E. Palm , and T. W. Concannon , 139–148. Springer. 10.1007/978-3-030-83028-1.

[infa70036-bib-0105] Shneidman, L. A. , M. E. Arroyo , S. C. Levine , and S. Goldin‐Meadow . 2013. “What Counts as Effective Input for Word Learning?” Journal of Child Language 40, no. 3: 672–686. 10.1017/S0305000912000141. 22575125 PMC3445663

[infa70036-bib-0106] Shrider, E. A. , and J. Creamer . 2023. Poverty in the United States: 2022 (Current Population Reports, No. P60‐280). U.S. Census Bureau, U.S. Department of Commerce. https://www.census.gov/content/dam/Census/library/publications/2023/demo/p60‐280.pdf.

[infa70036-bib-0107] Simmons, E. S. , and R. Paul . 2024. “Are Late Talkers Just Late? Neighborhood Density and Word Frequency Properties of Late Talkers' Spoken Vocabularies.” Journal of Speech, Language, and Hearing Research 67, no. 10: 3794–3802. 10.1044/2024_JSLHR-23-00769. PMC11482578 39302886

[infa70036-bib-0118] Singletary, B. , L. M. Justice , H. Jiang , W. G. Wilberforce , and D. Avelar . 2025. “Modeling the Mechanisms Through Which Conditions of Poverty Are Associated With Late Language Emergence in Young Children.” Early Childhood Research Quarterly 73: 121–134. 10.1016/j.ecresq.2025.07.003. PMC12530060 41113282

[infa70036-bib-0108] Sperry, D. E. , L. L. Sperry , and P. J. Miller . 2019a. “Language Does Matter: But There Is More to Language Than Vocabulary and Directed Speech.” Child Development 90, no. 3: 993–997. 10.1111/cdev.13125. 30102424

[infa70036-bib-0109] Sperry, D. E. , L. L. Sperry , and P. J. Miller . 2019b. “Reexamining the Verbal Environments of Children From Different Socioeconomic Backgrounds.” Child Development 90, no. 4: 1303–1318. 10.1111/cdev.13072. 29707767

[infa70036-bib-0110] Verganti, C. , C. Suttora , M. Zuccarini , et al. 2024. “Lexical Skills and Gesture Use: A Comparison Between Expressive and Receptive/Expressive Late Talkers.” Research in Developmental Disabilities 148: 104711. 10.1016/j.ridd.2024.104711. 38520885

[infa70036-bib-0111] Vernon‐Feagans, L. , M. Bratsch‐Hines , E. Reynolds , and M. Willoughby . 2020. “How Early Maternal Language Input Varies by Race and Education and Predicts Later Child Language.” Child Development 91, no. 4: 1098–1115. 10.1111/cdev.13281. 31317532 PMC6980228

[infa70036-bib-0112] Walters, C. E., Jr , R. Nitin , K. Margulis , et al. 2020. “Automated Phenotyping Tool for Identifying Developmental Language Disorder Cases in Health Systems Data (APT‐DLD): A New Research Algorithm for Deployment in Large‐Scale Electronic Health Record Systems.” Journal of Speech, Language, and Hearing Research 63, no. 9: 3019–3035. 10.1044/2020_JSLHR-19-00397. PMC7890229 32791019

[infa70036-bib-0113] Weber, J. M. , and E. Colunga . 2019. “Word‐Learning Biases Contribute Differently to Late‐Talker and Typically Developing Vocabulary Trajectories.” Proceedings of the Annual Meeting of the Cognitive Science Society 41. https://escholarship.org/uc/item/0cc2f37z.

[infa70036-bib-0114] Westerlund, M. , E. Berglund , and M. Eriksson . 2006. “Can Severely Language Delayed 3‐Year‐Olds Be Identified at 18 Months? Evaluation of a Screening Version of the MacArthur‐Bates Communicative Development Inventories.” Journal of Speech, Language, and Hearing Research 49, no. 2: 237–247. 10.1044/1092-4388(2006/020). 16671841

[infa70036-bib-0115] Wiig, E. H. , E. Semel , and W. A. Secord . 2013. Clinical Evaluation of Language Fundamentals – Fifth Edition (CELF‐5) [Measurement instrument]. Pearson.

[infa70036-bib-0116] Zimmerman, I. , V. Steiner , and R. Pond . 2011. Preschool Language Scale‐5 (PLS‐5): Examiner's Manual. Pearson.

[infa70036-bib-0117] Zubrick, S. R. , C. L. Taylor , M. L. Rice , and D. W. Slegers . 2007. “Late Language Emergence at 24 Months: An Epidemiological Study of Prevalence, Predictors, and Covariates.” Journal of Speech, Language, and Hearing Research 50, no. 6: 1562–1592. 10.1044/1092-4388(2007/106). PMC3521638 18055773

