# Supplementary material for: Examining Early Vocabulary Growth Trajectories in Late Talkers in a Low‐Income Longitudinal Sample
Source: Infancy. 2025 Aug 7;30(4):e70036. doi: 10.1111/infa.70036 (PMC12332339; doi:10.1111/infa.70036)
Supplement: Supplementary file 1 — Table S1 [file INFA-30-0-s002.docx]

Supplemental Table 1. *Two-level two-group linear model using English-speaking sample (N = 172): Log-transformed vocabulary growth trajectories for late talkers and peers.*

|  | Peers | | Late Talkers | |
| --- | --- | --- | --- | --- |
|  | *β* (S.E) | *p* | *β* (S.E) | *p* |
| Fixed Effects |  |  |  |  |
| Intercept at 8 months ^a^ | .87 (.17) | <.001 | .87 (.17) | <.001 |
| Age in months | .31 (.01) | <.001 | .15 (.01) | <.001 |
| Sex: 1 = Female | .36 (.15) | .013 | .22 (.19) | .252 |
| Maternal Education ^b^:  High school degree/GED vs no high   school degree/GED | .13 (.21) | .519 | .23 (.25) | .909 |
| College degree or higher vs no high   school degree/GED | .10 (.21) | .645 | -.04 (.24) | .870 |
| Random Effects |  |  |  |  |
| Level 1 | .62 (.05) | <.001 | .62 (.05) | <.001 |
| Level 2 | .44 (.06) | <.001 | .44 (.06) | <.001 |

*Note*. ^a^ With the subsample of 172 participants from English-speaking sample, we first compared multiple-group models with invariance trajectory , free slope, and free slope + free intercept. The free slope model was chosen because it provides the best fit among the three. Outcome is the log-transformed vocabulary size. Intercept of the model was set at 8 months old, or the youngest age when CDI was administered. Model fit: χ^2^ = 6.13, df = 7, *p* = .525. RMSEA = .000. CFI = 1.000. SRMR within = .018, SRMR between = .018. ^b^ The model does not compare High school degree/GED vs College degree or higher, because less than high school degree was used as the reference group. *β* = Unstandardized coefficient; S.E. = Standard error.
