# Supplementary material for: Examining Early Vocabulary Growth Trajectories in Late Talkers in a Low‐Income Longitudinal Sample
Source: Infancy. 2025 Aug 7;30(4):e70036. doi: 10.1111/infa.70036 (PMC12332339; doi:10.1111/infa.70036)
Supplement: Supplementary file 2 — Table S2 [file INFA-30-0-s001.docx]

Supplemental Table 2. *Words Produced scores for late talkers and peers at each time-point, and each month (8-30 months), using the MacArthur-Bates Communicative Development Inventory.*

|  | Late Talkers  (*TP4 n* = 58, 29%) | Peers  (*TP4 n* = 141, 71%) | Test of difference |
| --- | --- | --- | --- |
| Time-points |  |  |  |
| TP1 ^a^ (*n* = 123) | 3.50 (4.52) | 5.29 (7.25) | .098 |
| TP2 ^a^ (*n* = 166) | 8.88 (10.36) | 26.67 (42.51) | <.001 |
| TP3 ^b^ (*n* = 184) | 26.07 (28.57) | 168.17 (156.63) | <.001 |
| TP4 ^b^ (*n* = 199) | 41.21 (33.43) | 367.45 (186.55) | <.001 |
| Age in months |  |  |  |
| 8 months ^a^ | 1.54 (2.03) | 3.25 (3.27) | .048 |
| 9 months ^a^ | 4.09 (6.12) | 3.65 (3.36) | .827 |
| 10 months ^a^ | 3.00 (3.06) | 7.56 (9.67) | .052 |
| 11 months ^a^ | 6.80 (4.66) | 12.40 (15.47) | .475 |
| 12 months ^a^ | N/A | 10.00 (5.66) | N/A |
| 13 months ^a^ | 8.88 (10.70) | 28.91 (26.78) | .007 |
| 14 months ^a^ | 6.24 (7.66) | 21.60 (30.71) | <.001 |
| 15 months ^a^ | 10.80 (12.10) | 31.77 (72.59) | .166 |
| 16 months ^a^ | 5.50 (4.51) | 34.70 (23.36) | .003 |
| 17 months ^a^ | 26.00 (15.56) | N/A | N/A |
| 18 months ^b^ | N/A | 68.67 (40.27) | N/A |
| 19 months ^b^ | 21.88 (31.49) | 122.54 (123.18) | <.001 |
| 20 months ^b^ | 22.42 (15.35) | 201.33 (143.27) | <.001 |
| 21 months ^b^ | 30.75 (14.80) | 235.94 (219.12) | .002 |
| 22 months ^b^ | 29.00 (23.81) | 137.89 (133.17) | .042 |
| 23 months ^b^ | 38.11 (31.43) | 255.44 (174.66) | <.001 |
| 24 months ^b^ | 28.00 (26.32) | 341.29 (201.80) | <.001 |
| 25 months ^b^ | 60.38 (33.96) | 327.03 (171.38) | <.001 |
| 26 months ^b^ | 67.56 (43.39) | 325.11 (125.46) | <.001 |
| 27 months ^b^ | 31.50 (25.43) | 573.38 (157.71) | <.001 |
| 28 months ^b^ | 35.50 (26.16) | 497.45 (166.49) | <.001 |
| 29 months ^b^ | 27.33 (8.08) | 529.89 (170.54) | <.001 |
| 30 months ^b^ | N/A | 399.00 (115.28) | N/A |

*Note*. ^a^ Using the Communicative Development Inventory – Words and Gestures Form. ^b^ Using the Communicative Development Inventory– Words and Sentences Form. Mean values of CDI Word Produced raw scores are shown for each TP and age, and standard deviations are shown in parentheses. Mean and SDs were calculated based on available data so each month’s statistics are based on a different subsample depending on when each child was assessed. N/A means that not enough data are available to calculate the summary statistics. *Accompanies the paper entitled: “Examining Early Vocabulary Growth Trajectories in Late Talkers in a Low-Income Longitudinal Sample.”*
